# The influence of gut microbiota on the gut-brain-kidney axis and its implications for chronic kidney disease

**DOI:** 10.3389/fmicb.2025.1535356

**Published:** 2025-07-09

**Authors:** Jue Zhu, Yutong Fu, Chinasa Valerie Olovo, Jianguo Xu, Qian Wu, Wei Wei, Ke Jiang, Xueming Zheng

**Affiliations:** ^1^Department of Nephrology, People’s Hospital of Liyang, Liyang, China; ^2^School of Medicine, Jiangsu University, Zhenjiang, China; ^3^Danyang Hospital of Traditional Chinese Medicine, Danyang, China; ^4^Department of Microbiology, Faculty of Biological Sciences, University of Nigeria, Nsukka, Nigeria; ^5^Department of Urology, People’s Hospital of Liyang, Liyang, China; ^6^Institute of Digestive Diseases, Jiangsu University, Zhenjiang, China

**Keywords:** chronic kidney disease, gut-brain-kidney axis, gut microbiota, metabolites, therapy

## Abstract

The gut-brain-kidney axis represents a dynamic interplay among the gut microbiota, renal function, and neurological processes, emerging as a critical factor in chronic kidney disease (CKD) pathophysiology. This paper reviews recent data on the mechanisms and pathways that integrate gut-brain-kidney signaling and communication, advances in our understanding of this axis, and potential diagnostic and prognostic biomarkers and interventions for CKD. Literature search was conducted on PubMed, Scopus, Web of Science, and Embase using a combination of the keywords gut microbiota, gut microbiome, gut-brain axis, gut-kidney axis, gut-brain-kidney axis, chronic kidney disease, dysbiosis, therapy, metabolites, and neuroinflammation.” Relevant studies were selected and synthesized in this narrative review. Gut dysbiosis, characterized by microbial composition and function alterations, contributes to systemic inflammation and metabolic imbalances, exacerbating CKD progression. Uremic toxins such as indoxyl sulfate and p-cresyl sulfate, derived from microbial metabolism, impair kidney function and disrupt neurocognitive health via oxidative stress and neuroinflammation, highlighting the interconnectedness of these systems. Recent advances in high-throughput sequencing and metabolomics have elucidated mechanisms linking gut microbiota and associated metabolites to kidney and brain health, revealing the role of microbial diversity and metabolite profiles in disease outcomes. Studies demonstrate that probiotics, prebiotics, and dietary interventions targeting the gut microbiota can modulate systemic inflammation and reduce uremic toxin levels, offering therapeutic potential. Understanding the bidirectional signaling within the gut-brain-kidney axis opens avenues for novel biomarkers and interventions in CKD management.

## 1 Introduction

Chronic kidney disease (CKD) is a global health concern characterized by the progressive loss of kidney function over time. It is a complex condition with a multitude of etiologies, including diabetes, hypertension, glomerulonephritis, and polycystic kidney disease (Stevens et al., 2024). The disease affects approximately 10% of the global population, with prevalence varying across regions due to differences in age, demographics, socioeconomic status, and access to healthcare (Rovin et al., 2024) and studies have consistently demonstrated a rising prevalence in both developed and developing countries. This upward trend is largely driven by the aging population and the increasing incidence of risk factors like diabetes and hypertension (Rovin et al., 2024; Jadoul et al., 2024). As kidney function deteriorates, patients may experience fatigue, nausea, vomiting, loss of appetite, and difficulty concentrating (Liyanage et al., 2022). Furthermore, it is associated with an increased risk of cardiovascular disease, which is the leading cause of death among affected patients (Rovin et al., 2024) and other complications include anemia, bone disease, and electrolyte imbalances. Progression to end-stage kidney disease (ESKD) necessitates renal replacement therapy, such as dialysis or kidney transplantation, which imposes a significant financial burden on patients and healthcare systems (Lv and Zhang, 2019). As a result, early detection and comprehensive management are crucial for slowing disease progression and reducing the risk of complications. Disease management involves a multidisciplinary approach, including lifestyle modifications, medication, and specialized care (Rossing et al., 2022). Interestingly, the gut-brain-kidney axis has significant implications for the development and progression of CKD, offering options for the management and treatment of the condition.

In recent years, the gut-brain-kidney axis has emerged as a compelling framework for understanding the interconnected pathophysiology of CKD. This concept highlights the intricate, bidirectional communication among the gastrointestinal tract, the central nervous system (CNS), and the kidneys where dysfunction in one system can lead to cascading effects on the others (Yang et al., 2018). Data show concrete evidence of the existence of a bidirectional communication between the gut and brain (Mayer et al., 2022), gut and kidney (Evenepoel et al., 2017), and kidney and brain (Xie et al., 2022). This holistic perspective provides new insights into the pathogenesis of complex diseases and may lead to more integrated therapeutic approaches that consider the interdependence of the gut, brain, and kidneys (Yang et al., 2018). In renal impairment, this axis contributes to a feedback loop driven by gut dysbiosis, systemic inflammation, oxidative stress, blood pressure dysregulations, and neurocognitive decline (Bugnicourt et al., 2013; Anders et al., 2013).

Central to this axis is the gut microbiota, which plays a pivotal role in maintaining homeostasis across the gastrointestinal, neurological, and renal systems. The gut microbiota produces an array of bioactive metabolites, such as short-chain fatty acids (SCFAs), neurotransmitter precursors, and uremic toxins, that mediate communication between the gut, brain, and kidneys (Lin et al., 2025). Dysbiosis, an imbalance in microbial composition, has been associated with neuroinflammation, cognitive impairment, and kidney function decline, supporting the existence of a shared pathophysiological mechanism within this tri-organ axis. Dysbiosis and the accumulation of uremic toxins can trigger systemic inflammation and oxidative stress, which are key contributors to the progression of CKD and neurocognitive dysfunction (Anders et al., 2013; Cedillo-Flores et al., 2025). For example, studies in rat models of CKD showed that cognitive decline correlated with serum indoxyl sulfate levels and blood–brain barrier (BBB) disruption. Mechanistically, the authors reported that the uremic toxin, indoxyl sulfate, activates the aryl hydrocarbon receptor (AhR), and experiments using AhR knockout mice confirmed that AhR activation is a key driver of BBB damage (Bobot et al., 2020). Moreover, gut microbiota disturbances also influence blood pressure regulation in the gut-brain-kidney axis, a critical factor in progressive renal impairment. Dysbiosis in CKD can affect the production of metabolites such as SCFAs which has been shown to influence blood pressure through their effects on the autonomic nervous system and kidney function (Pluznick, 2016). Poorly regulated blood pressure in turn, accelerates kidney damage, creating another feedback loop where hypertension worsens the disease progression (Pluznick, 2016).

The interaction between immune components and the gut microbiota is pivotal in the function of the gut-brain-kidney axis, influencing the occurrence and development of other diseases such as inflammatory bowel diseases (IBD), vascular inflammation and cardiovascular diseases, tumors, obesity and metabolic syndrome, nervous system diseases, infectious diseases, and hepatic fibrosis (Wiredu Ocansey et al., 2023). Germfree (GF) mice subjected to kidney injury experience increased CD8 T-cell trafficking, inflammatory cytokine mediators, and worse disease course compared with normal wildtype (WT) mice. However, the conventionalization of GF mice with normal mouse stool leads to normalizing T-cell and NKT populations, and protection from kidney injury (Jang et al., 2009). Moreover, the gut microbiota and its associated metabolites promote the differentiation and function of anti-inflammatory macrophages, Treg cells, CD4 + CD8αα + regulatory cells, IL-10 + and/or IL-35 + B regulatory cells, as well as IL-22-producing innate lymphoid cells 3 (ILC3), which are involved in maintaining the gut mucosal homeostasis (Su et al., 2022) and protecting the kidneys (Su et al., 2022). Another key mechanism in the gut microbiota–immune system interaction is the participation of SCFAs such as acetate, butyrate, and propionate in the activation and ligation of various G protein–coupled receptors, including GPR109a, olfactory receptor-78, free fatty acid receptor 2, and free fatty acid receptor 3, which are crucial mechanisms by which the microbiota modulates immune cell function (Andrade-Oliveira et al., 2015; Noel et al., 2021).

Understanding this tri-organ communication opens new avenues for integrated management of renal disease. Thus, this study reviews the mechanisms and pathways associated with the gut-brain-kidney axis, advances in understanding this axis, and potential implications for CKD management and treatment. Current clinical studies and trials involving the gut-brain-kidney axis, as well as challenges and prospects in this field, are also examined.

## 2 The gut-brain-kidney axis: mechanisms and pathways

**Figure 1:**
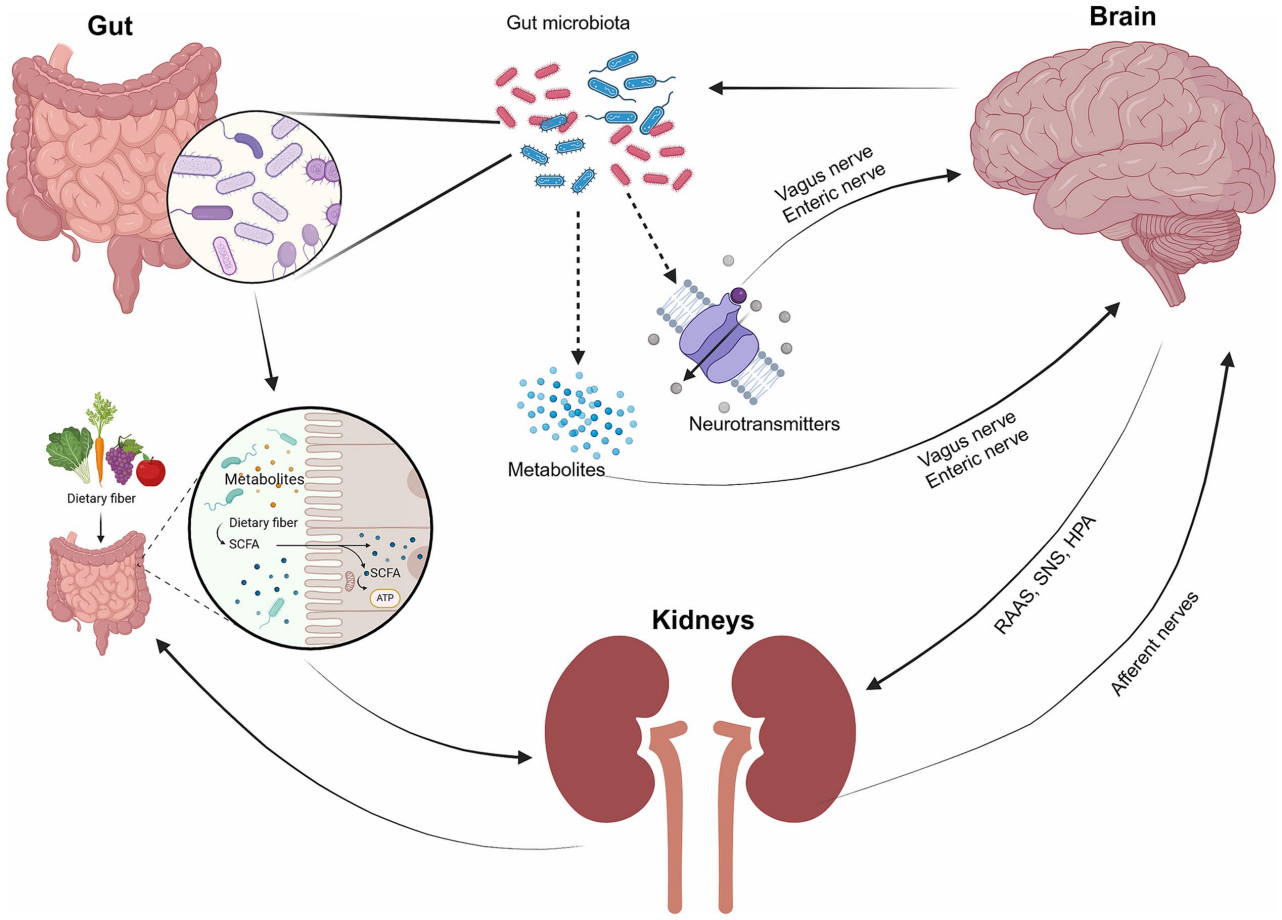
The interaction of the gut, brain, and kidneys. The gut microbiota serves as the key medium of communication between the gut, brain, and kidneys. The gut microbiota plays a crucial role in producing metabolites and neurotransmitters that impact brain function through interactions with the vagus nerve and the enteric nervous system. The gut also influences kidney function through metabolites, while the kidney interacts with the brain through hormonal pathways (e.g., RAAS, the SNS, the hypothalamic–pituitary–adrenal (HPA) axis, and afferent nerves).

The gut-brain-kidney axis involves intricate communication between the gastrointestinal tract, central nervous system (CNS), and kidneys through various mechanisms and pathways involving the gut microbiota. Key mechanisms include systemic communication, brain-kidney interactions, gut-kidney interactions, and integrated gut-brain-kidney signals (Figure 1). The gut microbiota plays a pivotal role, producing metabolites and neurotransmitters that influence brain function via the vagus nerve and enteric nervous system, while the hypothalamic–pituitary–adrenal (HPA) axis modulates stress responses, impacting both gut and brain functions (Yang et al., 2018; Hanscom et al., 2021; Vallianou et al., 2023). The brain regulates kidney function through the sympathetic nervous system (SNS) and hormonal pathways like the renin-angiotensin-aldosterone system (RAAS) (Stier et al., 2005). Conversely, the kidneys can signal the brain regarding their status, influencing homeostasis. Gut-kidney interactions involve the exchange of metabolites, including uremic toxins and oxalates, which affect kidney health (Mao et al., 2023; He et al., 2024). Additionally, inflammation and dysbiosis can lead to systemic effects, contributing to chronic conditions like CKD and neuroinflammation (Adesso et al., 2017). This interconnectedness highlights the potential for therapeutic interventions targeting one organ system to benefit the others.

### 2.1 Gut microbiota and systemic communication

The gut microbiota, a complex community of trillions of microorganisms residing primarily in the gastrointestinal tract, is crucial in maintaining systemic health. The microbiota is involved in numerous physiological processes, including digestion, immune modulation, and the production of essential vitamins and short-chain fatty acids (SCFAs) like butyrate, which contribute to intestinal barrier integrity and anti-inflammatory effects (Ahmed et al., 2022). Beyond the gut, the microbiota influences distant organs through the production of bioactive compounds that enter the bloodstream, affecting metabolic functions, immune responses, and even neurobehavioral processes (Romaní-Pérez et al., 2021). Disruptions in this microbial ecosystem, known as dysbiosis, have been linked to a range of diseases, including obesity, diabetes, cardiovascular disease, and mental health disorders like depression and anxiety, highlighting its critical role in systemic health (Zhao et al., 2023; Marano et al., 2023). For example, a study found that rotenone-induced microbiota dysbiosis is implicated in the pathogenesis of Parkinson’s disease via the microbiota-gut-brain axis, and fecal microbiota transplant (FMT) alleviates systemic inflammation, intestinal inflammation, and barrier destruction, attenuating BBB impairment and neuroinflammation. Mechanistically, the microbiota transplant reduced lipopolysaccharide (LPS) levels in the colon, the serum, and the substantia nigra (SN) of the midbrain, suppressing the toll-like receptor 4 (TLR4)/myeloid differentiation primary response 88 (MyD88)/nuclear factor kappa B (NF-κB) signaling pathway and its downstream pro-inflammatory products in both the SN and the colon (Zhao et al., 2021). In addition to the gut-brain and gut-kidney axes, the gut microbiota systemically communicates through other axes such as the gut-liver (Albillos et al., 2020), gut-heart (Zhao et al., 2023), gut-skin (Sinha et al., 2021), and gut-bone (Zhang et al., 2024) axes, among others.

### 2.2 Brain-kidney interactions

Patients with kidney dysfunction often experience multiple organ dysfunction, partly because the kidneys are connected to many other organs. There is a strong link between the kidneys and the brain, which can cause people with CKD to have a higher possibility of developing conditions like memory loss, stroke, and nerve damage (Lu et al., 2015). These anomalies occur because the kidneys and the brain share parts of their structure, have similar systems for controlling blood flow, and communicate through chemicals and other signals (Bugnicourt et al., 2013; Shiao et al., 2015). A study found that kidney afferent nerves stimulate a brain-kidney neural circuit in CKD and heart failure, whichpromotes the SNS to increase disease progression in both organs. The researchers indicate that in mouse models of CKD or heart failure, the overactivation of the kidney-brain neural circuit leads to increased sympathetic nerve activity targeting both the kidneys and the heart (Cao et al., 2023). Disrupting this pathway—either by severing kidney sensory nerves, selectively deleting the angiotensin II type 1a receptor (AT1a) in the subfornical organ (SFO), or using optogenetics to silence the kidney-SFO or SFO-paraventricular nucleus (PVN) connections—reduces sympathetic output, which in turn diminishes structural damage and functional impairment in the kidneys and heart in these models of CKD and heart failure (Cao et al., 2023). This suggests novel therapeutic targets for CKD or heart failure through this kidney-brain neural circuit.

Evidence suggests that patients with CKD are prone to increased incidence of ischemic strokes, with cognitive dysfunction, dementia, transient infarcts, and white matter lesions widespread in mild to moderate CKD patients (Chelluboina and Vemuganti, 2019). Moreover, lower renal function correlates with less effective dynamic cerebral autoregulation in acute ischemic stroke, predicting a bad outcome (Castro et al., 2018). The brain also influences kidney function through several key pathways, primarily involving neural, metabolic, and hemodynamic mechanisms, including the SNS, RAAS, HPA axis, and neural circuits and associated feedback loops (Freeman and Wadei, 2015; Dulam et al., 2024). These observations indicate the intricate connection between the brain and kidneys in health and disease.

### 2.3 Gut-kidney interactions

The gut-kidney axis is an emerging field of study that highlights the bidirectional communication between these two organs, with the gut microbiota playing a significant role in their function and disease. Disruption of the normal gut microbiota may lead to intestinal dysbiosis, barrier dysfunction, bacterial translocation, and excessive production of uremic toxins, including indoxyl sulfate (IS), p-cresyl sulfate (p-CS), and trimethylamine-N-oxide (TMAO), all of which are implicated in the variant processes of kidney diseases development (Chen et al., 2019). Similarly, the changes accompanying renal failure will likely influence the gut microbiota or the ecosystem of microorganisms resident in the intestine (Bartochowski et al., 2022). For example, CKD patients have been found to exhibit increased intestinal permeability, reduced gut motility, bacterial overgrowth, bacterial translocation, and intestinal inflammation (Cosola et al., 2021; Strid et al., 2003). Moreover, patients with ESKD show an expansion of proteolytic bacteria involved in uremic toxins’ metabolism but a reduced relative abundance of carbohydrate-fermenting bacteria such as *Lactobacillacea*e and *Prevotellaceae* families (Wong et al., 2014). Further analysis indicated that among the 19 overgrown bacterial families, 12 possessed the urease gene (*Alteromonadaceae*, *Clostridiaceae*, *Cellulomonadaceae*, *Enterobacteriaceae*, *Dermabacteraceae*, *Halomonadaceae*, *Micrococcaceae*, *Moraxellaceae*, *Methylococcaceae*, *Pseudomonadaceae*, *Polyangiaceae*, and *Xanthomonadaceae*), 5 harbored the uricase gene (*Dermabacteraceae*, *Cellulomonadaceae*, *Xanthomonadaceae*, *Micrococcaceae*, and *Polyangiaceae*) producing ammonia that is harmful to the intestinal epithelium, and 3 expressed the tryptophanase gene necessary for the conversion of tryptophan into indole, which is then metabolized by the liver into uremic toxins (*Enterobacteriaceae*, *Clostridiaceae*, and *Verrucomicrobiaceae*) (Wong et al., 2014). Other studies report that the concentration of SCFAs, which is associated with a decreased abundance of *Faecalibacterium, Enterococcus, Enterobacter, Bifidobacterium, Clostridium, Bacteroides,* and *Roseburia*, is inversely correlated with the degree of renal insufficiency in patients (Wang et al., 2019; Felizardo et al., 2019).

Inflammation is a common link between gut health and kidney disease. The gut microbiota can influence systemic inflammation, which in turn affects kidney function (Onal et al., 2019). Dysbiosis has been associated with an increased permeability of the gut barrier, a condition that allows endotoxins like LPS to enter the bloodstream, triggering an inflammatory response that can contribute to the progression of CKD (Evenepoel et al., 2023). Furthermore, inflammation in the gut has been linked to an increase in circulating pro-inflammatory cytokines, which are known to contribute to kidney damage and fibrosis (Lau et al., 2015). Thus, the gut-kidney axis represents a complex and significant interaction between these two organs, with gut microbiota playing a central role in kidney health and disease. Dysbiosis, inflammation, and gut-derived metabolites are all critical factors in the progression of kidney disease.

### 2.4 Integration of gut, brain, and kidney signals

**Figure 2:**
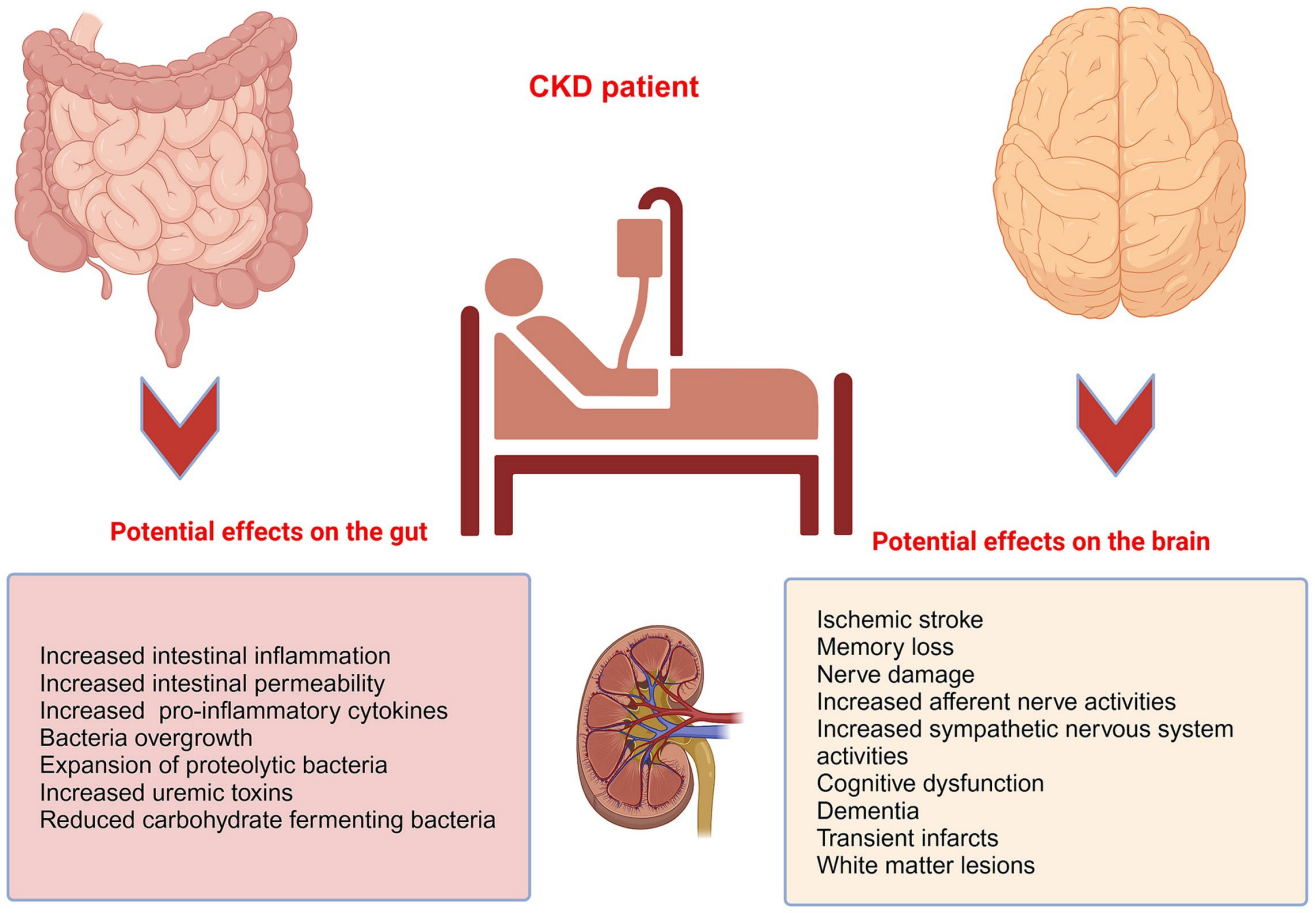
The pathological implications of CKD on the gut and the brain. CKD patients are prone to a higher propensity of pathological imparts on the gut and the brain. For example, patients with CKD often display heightened intestinal permeability, decreased gut motility, bacterial overgrowth, translocation of bacteria, and intestinal inflammation, and are prone to increased incidence of ischemic strokes, with cognitive dysfunction, dementia, transient infarcts, and white matter lesions.

The gut-brain-kidney axis is closely linked to the development and progression of several specific diseases, with each organ system influencing the others in ways that can exacerbate or mitigate these conditions, including CKD (Figure 2), hypertension, irritable bowel syndrome, and kidney stones. The complex regulation of gastrointestinal function is mediated by the autonomic (ANS) and enteric nervous systems (ENS). The ANS transmits physiological signals from the gut, including acidity, nutrient levels, osmolarity, and pain, to the CNS. The ENS, comprising the myenteric and submucosal plexuses, facilitates local neural communication within the intestinal tract and integrates with the autonomic nervous system (Dowling et al., 2022; Margolis et al., 2021). The brain again plays a crucial role in regulating kidney function, ensuring optimal renal blood flow, glomerular filtration rate, acid–base balance, and electrolyte balance through the ANS and the endocrine system (Afsar et al., 2016a). The kidney, in turn, maintains brain function by regulating blood volume, pressure, and levels of electrolytes, such as sodium, potassium, and chloride, which are essential for nerve and muscle function, including those in the brain (Dalal et al., 2024). The vagus nerve serves as a critical communication pathway, transmitting gut-derived signals to both brain and kidney tissues, and its activation modulates renal sympathetic tone and inflammatory responses (Keefer, 2018). Moreover, gut microbes produce neuroactive compounds such as SCFAs and tryptophan metabolites that influence brain function and renal physiology. These metabolites regulate the HPA axis and renal sodium movement (Dalile et al., 2019). Thus, the disruptions of the kidney-brain axis contribute to the high morbidity of neurological disorders, such as cognitive impairment in CKD (Yan et al., 2024).

The gut microbiota constantly communicates with vital organ systems of the host, such as the brain (Dinan and Cryan, 2017), kidney (Bartochowski et al., 2022), autonomic nervous system (ANS) (Bercik et al., 2011), and immune system (Rooks and Garrett, 2016). For example, a study found that *Bifidobacterium longum* NCC3001 normalizes anxiety-like behavior and hippocampal brain-derived neurotrophic factor (BDNF) in mice with infectious colitis. Moreover, *Bifidobacterium longum* decreases the excitability of enteric neurons and signals to the CNS by activating vagal pathways at the level of the enteric nervous system (Bercik et al., 2011). Fecal *Lactobacillus mucosae* NK41 and *Bifidobacterium longum* NK46 alleviate anxiety/depression and colitis by suppressing gut dysbiosis via downregulating hippocampal NF-κB activation, BDNF expression, Iba1 + cell population, and blood corticosterone, IL- 6, TNF-α, and LPS levels (Han and Kim, 2019). On the other hand, gut dysbiosis triggers systemic inflammation through LPS and cytokine release, affecting BBB integrity and renal function. This triad also shares common inflammatory pathways mediated by TLR4 activation (Zhao et al., 2021; Zhou X. et al., 2024). Thus, gut, brain, and kidney signals, a tripartite communication network, are integrated through multiple signaling mechanisms involving vagal signaling, microbiota-derived metabolites, immune-mediated crosstalk, and neuroendocrine regulation.

## 3 Advances in understanding the gut-brain-kidney axis

### 3.1 Microbial metabolites and their impact

Recent studies are increasingly demonstrating the influence of gut microbial-derived metabolites in the pathophysiology of the brain and kidneys. For example, SCFAs regulate inflammation, oxidative stress, and fibrosis and have been involved in kidney disease by activating the gut-kidney axis (Li et al., 2017; He et al., 2024). SCFAs exert their effects by activating transmembrane G protein-coupled receptors and inhibiting histone acetylation (Li et al., 2017). In assessing the complex interconnection of gut metabolites with renal and cerebrovascular endothelial dysfunction, proximal tubule dysfunction, and podocyte injury, a study found that metabolites belonging to the retinoic acid signaling and nitrogen metabolic pathways could differentiate normoalbuminuria (P1) from microalbuminuria (P2) and macroalbuminuria (P3) patients. Moreover, tyrosine, IS, phenylalanine, serotonin sulfate, and all-trans retinoic acid were highlights of the metabolic fingerprint of the P1 group vs. P2, P3, and the healthy control groups (Balint et al., 2023a). Similarly, metabolites potentially derived from gut microbiota were reported to be associated with renal and cerebrovascular endothelial, podocyte, and proximal tubule damage in early diabetic kidney disease in diabetes Mellitus patients (Balint et al., 2023b). This indicates that metabolite profiling of the gut-renal-cerebral axis reveals a particular pattern in different stages of kidney diseases. Arginine, hippuric acid, dimethylarginine, butenoylcarnitine, indoxyl sulfate, sorbitol (in serum), and p-CS (in urine) served as possible biomarkers for early diabetes kidney disease (Balint et al., 2023c). These observations indicate that quantitative, targeted analysis of gut microbiota-derived metabolites provides novel biomarkers for early kidney diseases and CKD, thus deserves further exploration. Similar findings are reported of gut microbes as changes in their composition are associated with CKD. For example, compared with healthy individuals, culturable anaerobic bacteria is reduced in the feces of patients with stage 3–4 CKD (Ranganathan et al., 2009). By contrast, an elevated abundance of culturable aerobic bacteria is reported in the feces of patients with CKD who were not yet on dialysis compared with healthy adults (Yang et al., 2018). *Faecalibacterium prausnitzii* is a prominent butyrate-producing bacterium within the human gut microbiota (Sokol et al., 2008). Butyrate, an SCFA, is known for its anti-inflammatory properties and its role in maintaining intestinal barrier integrity (Diep et al., 2025). In CKD, studies have shown a significant depletion of *F. prausnitzii* in patients, correlating with disease progression (Jiang et al., 2016). This depletion leads to reduced butyrate levels, which can compromise gut barrier function and promote inflammation, thereby exacerbating renal injury. Recent research has demonstrated that supplementation with *F. prausnitzii* in CKD mouse models resulted in improved renal function by reducing renal inflammation and lowering the serum levels of various uremic toxins, which was partly attributed to the butyrate-mediated GPR-43 signaling in the kidney (Li et al., 2022).

In the interaction with host intestinal tissues, the gut microbiota influences brain functions, and microbial dysbiosis has been implicated in brain disorders such as Alzheimer’s disease (AD) and neuropsychiatric conditions (Wang W. et al., 2022). L-tryptophan metabolites and SCFAs are key signaling molecules in the gut microbiota-brain axis. The primary tryptophan metabolites the microbiota produces include indole derivatives such as indole-3-pyruvic acid, indole-3-acetaldehyde, and IS (Wang W. et al., 2022; Salminen, 2023). In the intestinal host cells, indoleamine and tryptophan 2,3-dioxygenases (IDO/TDO) activate the kynurenine (KYN) pathway, generating KYN metabolites, many of which activate aryl hydrocarbon receptor (AhR) signaling. In cases of CKD, elevated serum levels of IS contribute to AD development by disrupting the BBB and impairing cognitive functions (Salminen, 2023). Moreover, the gut microbiota affects behavior, regulates neurotransmitter production in the gut and brain, and plays a role in brain development and myelination patterns (Bolyen et al., 2019; Teubner et al., 2007). A recent study revealed that a gut-derived molecule influences complex behaviors in mice through effects on oligodendrocyte function and myelin patterning in the brain. The findings indicate that microbial metabolite 4-ethylphenyl sulfate (4EPS) enters the brain and is associated with changes in region-specific activity and functional connectivity, including altered oligodendrocyte function, impaired oligodendrocyte maturation, and decreased oligodendrocyte-neuron interactions (Needham et al., 2022). Interestingly, mice exposed to 4EPS exhibited anxiety-like behaviors, and treatments that promote oligodendrocyte differentiation were able to prevent the behavioral effects caused by 4EPS (Needham et al., 2022). These observations highlight the complex interaction within the gut-brain-kidney axis, offering potential for diagnostics and therapeutics in CKD.

### 3.2 Neuroinflammation and CKD

CKD is increasingly recognized as a condition that affects not only the kidneys but also has systemic effects, including on the CNS, often contributing to neuroinflammation. One of the significant mechanisms by which CKD influences brain health is through gut-derived signals, including uremic toxins and microbial metabolites. A key study by Andrade-Oliveira et al. (2015) investigated the gut-brain-kidney axis and revealed that CKD increases gut permeability, leading to the translocation of bacteria and their products, such as LPS, into the bloodstream. This translocation activates toll-like receptors (TLRs), including TLR4, in microglial cells within the brain, triggering neuroinflammatory responses. The study demonstrated elevated levels of pro-inflammatory cytokines, such as IL-1β and TNF-α, in the brains of CKD mice, indicating that gut-derived signals play a central role in mediating neuroinflammation in CKD (Andrade-Oliveira et al., 2015). Adesso and his colleagues further explored the contribution of gut-derived metabolites, mainly IS, a uremic toxin produced by gut bacteria. The researchers found that elevated IS levels in CKD patients correlate with oxidative stress and neuroinflammation, contributing to the deterioration of glial cells. IS increased the expression of inducible nitric oxide synthase and cyclooxygenase-2 (COX-2), TNF-α, IL-6, and nitrotyrosine formation in primary mouse astrocytes and mixed glial cells (Adesso et al., 2017). A recent study found that gut bacterial metabolite, Ruminococcaceae isoamylamine (IAA), which is enriched in older adults and aged mice, promotes age-related cognitive dysfunction by promoting microglial cell death (Teng et al., 2022). Microglia, tissue-resident macrophages of the central nervous system (CNS), play an essential role in the monitoring and intervening synaptic and neuron-level activities (Wang M. et al., 2022). Mechanistically, IAA promotes apoptosis of microglial cells by recruiting the transcriptional regulator p53 to the S100A8 promoter region (Teng et al., 2022). This disruption and subsequent infiltration of inflammatory mediators are associated with cognitive impairment in CKD patients, providing further evidence of a gut-brain connection (Sun et al., 2021). Vaziri and colleagues identified SCFAs as crucial regulators of neuroinflammation in CKD. Their research demonstrated that SCFA-producing bacteria are reduced in CKD, resulting in lower circulating levels of SCFAs. This depletion impairs the activation of G-protein-coupled receptors and histone deacetylation, which are essential for regulating brain inflammatory responses. The study concluded that the loss of SCFAs contributes to an imbalance in pro- and anti-inflammatory pathways in the CNS, exacerbating neuroinflammation in CKD (Vaziri et al., 2013). In addition to SCFAs and IS, p-CS, another gut-derived uremic toxin, has been implicated in CKD-related neuroinflammation (Meijers and Evenepoel, 2011).

According to Rysz and colleagues, as CKD progresses, protein-bound uremic toxins, such as IS, p-CS, p-cresyl glucuronide, and indole-3-acetic acid (IAA), gradually accumulate. The condition may also trigger intestinal inflammation and damage to the epithelial barrier, accelerating the systemic translocation of bacteria-derived uremic toxins. This leads to oxidative stress, negatively impacting the kidneys, cardiovascular system, and endocrine functions (Rysz et al., 2021). Furthermore, studies reveal that KYN pathway metabolites, particularly quinolinic acid (QA), are significantly elevated in CKD patients due to altered tryptophan metabolism by gut microbiota. QA is a potent neurotoxin that promotes neuroinflammation by activating N-methyl-D-aspartate (NMDA) receptors in neurons, leading to excitotoxicity (Connell et al., 2022; Hui et al., 2023). QA accumulation is associated with brain inflammation and cell death, and patients with advanced CKD exhibit elevated plasma KYN metabolites and QA, which uniquely correlate with fatigue and reduced quality of life (Saliba et al., 2024), supporting the role of gut-derived KYN metabolites in linking CKD to neuroinflammation. Moreover, studies show a widespread reduction in acetylcholinesterase activity, decreased neuronal arborization and dendritic spine density in specific brain regions of CKD mice. Oxidative stress, inflammation, and mitochondrial dysfunction were identified in these brain regions, suggesting they are the root causes of the observed neurochemical and histopathological changes (Mazumder et al., 2019). These results are significant in understanding the crosstalk within the gut-brain-kidney axis and offer therapeutic insights for managing neurological complications associated with CKD.

Together, these studies highlight the complex interplay between CKD, neuroinflammation, and gut-derived signals, providing strong evidence that the gut microbiota and its metabolites play a significant role in promoting neuroinflammatory processes in CKD, which in turn contribute to cognitive decline and neurodegenerative changes.

### 3.3 The role of stress and mental health

Chronic stress and mental health disorders such as depression, cognitive impairment, psychological distress, and anxiety have been increasingly implicated in the progression and worsening of CKD, with the gut-brain axis playing a central role in this connection (Gela et al., 2024). The bidirectional communication between the gut, brain, and kidneys suggests that stress-induced changes in gut microbiota, inflammatory processes, and neural pathways can directly influence kidney function. In one study, dysbiosis in individuals exposed to chronic stress was linked to increased intestinal permeability, also known as “leaky gut.” This permeability allows bacterial endotoxins to enter the bloodstream, promoting systemic inflammation. The increased inflammatory response exacerbates kidney damage in CKD patients, showing how chronic stress contributes to the progression of kidney disease through gut-derived signals (Zhou T. et al., 2024). Additionally, stress-induced activation of the HPA axis can worsen inflammation and dysbiosis, further impacting kidney function (Zhou T. et al., 2024). According to Palmer and colleagues, depression is linked to elevated serum cortisol, which worsens albuminuria and CKD progression (Turin et al., 2013). Mental health disorders like depression also play a role in CKD progression. Research demonstrates that depression can influence gut microbiota composition, increasing the risk of both systemic inflammation and CKD progression. In one study, depressive symptoms in CKD patients were associated with elevated oxidative stress and inflammatory responses leading to poorer clinical outcomes (Zhou T. et al., 2024). Additionally, anxiety and stress trigger SNS hyperactivity, increasing renin release and angiotensin II, which promote renal vasoconstriction and tubulointerstitial damage (Wang and Dong, 2016) while elevated norepinephrine levels under mental distress accelerating kidney function decline (Tidgren and Hjemdahl, 1989). Moreover, the KYN pathway, which is altered in both depression and CKD, has been implicated in gut permeability and immune dysregulation, further linking the gut-brain axis with kidney dysfunction (Kearns, 2024).

By disrupting gut microbial balance, compromising barrier integrity, and amplifying inflammatory signaling, chronic stress and mental health disorders exacerbate kidney dysfunction in CKD patients (Drew et al., 2019; Buoli et al., 2024). The findings highlight a strong connection between the gut-brain axis, chronic stress, mental health disorders, and CKD, indicating that addressing mental health and gut microbiota changes may be important for managing CKD progression. Moreover, therapeutic strategies targeting the gut-brain axis, including interventions aimed at restoring gut microbial composition, metabolite balance, and reducing inflammation, could be beneficial in mitigating CKD progression associated with chronic stress and mental health issues.

## 4 Potential biomarkers from gut microbiome profiling and metabolomics

The many microorganisms that make up the gut microbiota, such as the viruses, fungi, and bacteria, significantly impact the communication of the gut-brain-kidney axis (Yang et al., 2018), and the metabolites they produce, including neurotransmitters and SCFAs, can affect brain and kidney function (Dicks, 2022; Magliocca et al., 2022). Reports from recent studies reveal that gut microbial dysbiosis is an essential contributor to CKD pathophysiology and neurodegenerative conditions, making it a potent target for both early diagnosis of the disease and use in personalized treatment (Amini Khiabani et al., 2023; Kandpal et al., 2022). As a result, technological advances in microbiome profiling, such as metagenomic analyses and 16S ribosomal RNA sequencing, have facilitated the detailed characterization of microbial communities in CKD patients. Gut microbiota plays a very crucial role in the progression of CKD. Such roles, which include uremic toxins production, alteration of metabolic pathways, and modulation of inflammation and oxidative stress, influence CKD pathogenesis (Pires et al., 2024). Metabolomics, on the other hand, provides a comprehensive view of metabolic changes in CKD patients by analyzing various metabolites in biological fluids. Profiling metabolites associated with the gut-brain-kidney axis, such as uremic toxins, can offer insights into disease mechanisms and identify biomarkers for early diagnosis and disease monitoring (Yang et al., 2018).

Bioinformatics analysis of data sourced from the National Center for Biotechnology Information (NCBI) database revealed that beyond the increased F/B (Firmicutes/Bacteroides) ratio of CKD patients compared to the healthy control, there is an increased abundance of bacteria closely associated with kidney impairment in the CKD group, including *Ralstonia* and *Porphyromonas* which are known to correlate negatively with estimated glomerular filtration rate (eGFR) and may serve as markers of CKD progression (Liu W. et al., 2023). The systematic review of Voroneanu et al. (2023) revealed that the gut microbiota of CKD patients, even at the early stages of the disease, show some distinct alterations in relation to those of the healthy controls. *Ruminococcus* and *Roseburia* greatly differentiated CKD patients from healthy controls, with *Roseburia* showing a constant decline in CKD patients, especially those with ESKD (Voroneanu et al., 2023). With the aid of shotgun sequencing of fecal samples and targeted metabolomics profiling of serum samples in CKD patients of different stages and non-CKD controls, Wu et al. (2020a) observed that while the species abundance of *Alloscardovia omnicolens*, *Merdibacter massiliensis*, and *Clostridium glycyrrhizinilyticum* showed significant increase as CKD progressed, *Prevotella* sp. 885, *Weissella confuse*, *Roseburia faecis*, and *Bacteroides eggerthii* were found to decrease profoundly with CKD progress. Certain species also showed significant alterations in specific CKD stage(s). *Cetobacterium somerae* and *Candidatus Stoquefichus* sp. KLE1796, were associated with mild CKD (stages 1 and 2), *Fusobacterium mortiferum*, *Bariatricus massiliensis*, and *Bacteroides stercorirosoris*, were linked to moderate CKD (stage 3), and *Merdimonas faecis* involved with advanced CKD (stages 4 and 5). At the metabolomics level, the fatty acid biomarkers identified include capric acid, caproic acid, propionic acid, and heptanoic acid and showed a significant reduction in at least one of the severity groups listed above, compared to the healthy controls and the concentration of the uremic toxins IS and p-CS were significantly high at the advanced stage. Decreased abundance of *Prevotella* sp. 885 was associated with urea excretion, while increased level of p-CS and decreased level of caproic acid were negatively and positively correlated with eGFR, respectively. The interconnectedness between circulating microbial metabolites was also demonstrated to be interconnected with gut microbial species at different CKD stages. Moreover, while microbial genes associated with secondary bile acid biosynthesis showed differential abundance at the early stage, pathways related to LPS biosynthesis and lipid metabolism were abundant at the advanced stage (Wu et al., 2020a). This further validates the importance of gut microbiota and metabolites in kidney health. Application of 16S rRNA gene sequencing in another study revealed that seven bacteria genera (*Escherichia*_*Shigella*, *Dialister*, *Lachnospiraceae*_ND3007_group, *Pseudobutyrivibrio*, *Roseburia*, *Paraprevotella*, and *Ruminiclostridium*) and two species (*Collinsella stercoris* and *Bacteroides eggerthii*) were identified as key-CKD associated microbiota strongly correlated across CKD stages. For example, significant enrichment of the *Escherichia*_*Shigella* genus was strongly associated with advances in CKD, while the increased abundance of *Pseudobutyrivibrio*, *Roseburia*, *and Ruminoclostridium* spp. *Dialister* and *Lachnospiraceae*_ND3007_group were negatively correlated with CKD severity. Interestingly, *Paraprevotella* (AUC, 0.78), *Pseudobutyrivibrio* (AUC, 0.76), and *Collinsella stercoris* (AUC, 0.83) showed more superiority than the usual urine protein/creatinine ratio (AUC, 0.755) in discriminating CKD patients from healthy controls even at early disease stage. Furthermore, the levels of IS and p-CS increased as CKD progressed, reflecting the extent of kidney damage. The findings from the study were validated in a secondary cohort (Wu et al., 2020b).

The study of Wang and colleagues suggests that imbalances in CKD-related gut microbiota and metabolite pathways may accelerate disease progression and offer potential for early diagnostic and therapeutic interventions. They found that out of 26 microbial species that changed in CKD patients, 18 species altered with disease progression (increase in the relative abundance of *Citrobacter freundii*, *Citrobacter werkmanii*, *Flavonifractor plautii*, and *Anaerostipes caccae* and decrease in the relative abundance of *Methanobrevibacter smithii*, *Coprococcus comes*, *Coprococcus eutactus*, *Clostridium sporogenes*, *Ruminococcus callidus*, *Ruminococcus bromii*, *Roseburia hominis*, *F. prausnitzii*, *Veillonella parvula*, *Megasphaera elsdenii*, *Dialister succinatiphilus*, *Acidaminococcus intestini*, *Faecalicoccus pleomorphus*, and *Subdoligranulum* var*iabile*) while 8 species were specific to particular CKD stages (Increased level of *Megasphaera micronuciformis* in the mild CKD group, decreased expression of *Alistipes indistinctus*, *Alistipes inops*, and *Bacteroides uniformis* in the moderate CKD group, and increased abundance of the *Turicibacter sanguinis* with a concomitant reduction in the levels of *Streptococcus mutans*, *Bifidobacterium adolescentis*, and *Lactobacillus crispatus* in the ESKD group). In metabolomics, significant alterations were observed in metabolism related to arginine and proline, arachidonic acid, glutathione metabolism, and ubiquinone and other terpenoid-quinone biosynthesis pathways as CKD progressed with further metabolomic analyses revealing that variations in the distributions of pro-oxidant and toxic metabolites from the four identified metabolic pathways were observed in the feces and serum of the patients as the disease progressed. Whereas the levels of 1,2-benzoquinone, 11-dehydro-thromboxane B2, 12-KETE, L-malic acid, N2-succinyl-L-ornithine, ornithine, argininosuccinic acid from the fecal metabolites gradually decreased as CKD progressed, chlorohydroquinone, hydroquinone, L-cystine, fecal 12-keto-tetrahydro-leukotriene B4 (12-keto-tetrahydro-LTB4), and L-cysteine levels gradually increased Integrated network analysis based on metabolomics and metagenomics data revealed that *Ruminococcus bromii*, fecal hydroquinone, and serum creatinine were the major contributors to this network, therefore play major roles in CKD progression. Furthermore, with the aid of the non-invasive diagnostic model based on the combination of gut microbial species and fecal metabolites, the authors developed a model incorporating *R. bromii*, fecal hydroquinone, L-cystine, and 12-keto-tetrahydro-LTB4 in the differentiation of mild, moderate, and advanced CKD patients from healthy controls. This model was demonstrated to attain an area under the curve (AUC) of >0.9 for classifying CKD severity, outperforming serum creatinine for detecting mild CKD (AUC: 0.972 vs. 0.896) (Wang et al., 2023). Another study demonstrated that while branched-chain amino acids (valine, leucine, and isoleucine) and taurine were significantly decreased, three metabolites of the kynurenine pathway, specifically 2-aminobenzoic acid, xanthurenic acid, and hydroxy picolinic acid were reported to be significantly upregulated in ESKD patients compared to CKD patients. Receiver Operating Characteristic (ROC) analysis revealed that vanillic acid and N-hydroxy-isoleucine were the highest discriminative metabolites with an AUC value of 1 and also 2-aminobenzoic acid and picolinic acid showed high discriminative ability with an AUC of 0.995 and these might serve as potential prognostic biomarkers to monitor the progression of CKD to ESKD (Dahabiyeh et al., 2023). The team of Chen successfully identified OTUs or metabolites that clearly differentiate distinct CKD groups (diabetic d-CKD, hypertensive H-CKD, and CKD with no comorbidity NC-CKD) from healthy participants. Significant associations between *Streptococcus*, *Clostridium*, *Culturomica*, and *Bacteroides* genera and four NC-CKD-enriched metabolites, including Arachidonic acid, L-Phenylalanine, Dihomogamma-linolenic acid, and N-Acetylputrescine were identified. Again, distinct correlations of *Fusobacterium* genera, *Megasphaera elsdenii*, *Ruminococcus gnavus*, and *Lactobacillus* genera with L-Proline and Stearic acid were identified to discriminate the d-CKD patients from the healthy participants. The close associations of relatively abundant Stearic acid, Amiloride, and 3,4-Dimethoxyphenylethylamine with the identified OTUs, including *Escherichia marmotae*, *Enterobacter hormaechei*, *Shigella boydii*, *Citrobacter koseri*, and *Subdoligranulum* var*iabile* notably separated h-CKD groups from healthy controls. More importantly, these identified specific OTU-metabolite associations show strong discrimination between healthy controls and CKD patients with ROC analyses. These specific species-metabolite associations clearly differentiated NC-CKD, d-CKD, and H-CKD patients from healthy controls with an AUC value of 0.962, 0.913, and 0.901, respectively. These findings indicate the potential relevance between species-OTU and metabolite associations in diagnosing CKD with distinct pathogenic factors (Chen T. H. et al., 2023).

These reports, summarized in Table 1, provide sound evidence of the contributions of the gut microbiota and associated metabolites in CKD. Since certain of these biomarkers can serve as early diagnostic biomarkers for CKD, complications that arise as the disease progresses, such as cardiovascular diseases and cognitive impairment, may be countered as early detection could lead to early management and delayed progression of kidney function decline.

**Table 1:** Biomarkers from the gut microbiota/metabolites and relevance in CKD.

S/N	Biomarker	Finding	Methodology	Prognostic potential	References
1	Increase in the abundance of *Ralstonia*, *Porphyromonas*	Negative correlation with eGFR	Bioinformatics analysis	Yes	Liu W. et al. (2023)
2	*Ruminococcus* and *Roseburia* decline	Differentiates CKD patients from healthy controls	Systematic review	Yes	Voroneanu et al. (2023)
3	Increased abundance of *Alloscardovia omnicolens*, *Merdibacter massiliensis*, and *Clostridium glycyrrhizinilyticum*	Differentiates CKD patients from healthy controls	Shotgun sequencing of fecal samples	Yes	Wu et al. (2020a)
4	Decrease in the abundance of *Prevotella* sp. 885, *Weissella confusa*, *Roseburia faecis*, and *Bacteroides eggerthii*	Differentiates CKD patients from healthy controls	Shotgun sequencing of fecal samples	Yes	Wu et al. (2020a)
5	Presence of *Cetobacterium somerae* and *Candidatus Stoquefichus* sp. KLE1796 Increase in *Megasphaera micronuciformis*	Associated with mild CKD	Shotgun sequencing of fecal samples	No	Wu et al. (2020a), Wang et al. (2023)
6	Increased levels of *Fusobacterium mortiferum*, *Bariatricus massiliensis*, and *Bacteroides stercorirosoris* Decreased levels of *Alistipes indistinctus*, *Alistipes inops*, and *Bacteroides uniformis*	Linked to moderate CKD	Shotgun sequencing of fecal samples	No	Wu et al. (2020a), Wang et al. (2023)
7	Presence of *Merdimonas faecis* Increased abundance of *Turicibacter sanguinis*; decrease in abundance of *Streptococcus mutans*, *Bifidobacterium adolescentis*, and *Lactobacillus crispatus*	Implicated in advanced CKD/ESKD	Shotgun sequencing of fecal samples	No	Wu et al. (2020a), Wang et al. (2023)
8	IS, p-CS	Increased concentration as CKD progresses	Targeted metabolomics profiling of serum samples	Yes	Wu et al. (2020a)
9	Differential abundance of microbial genes associated with secondary bile acid biosynthesis	Associated with early CKD	Shotgun sequencing and metabolomics profiling	Yes	Wu et al. (2020a)
10	Increase in pathways related to LPS biosynthesis and lipid metabolism	Associated with advanced CKD	Shotgun sequencing and metabolomics	Yes	Wu et al. (2020a)
11	Increased abundance of *Escherichia*_*Shigella*	Associated with advanced CKD	16S rRNA gene sequencing	Yes	Wu et al. (2020b)
12	Reduction in the relative abundance of *Pseudobutyrivibrio*, *Roseburia*, *Ruminoclostridium* spp. *Dialister* and *Lachnospiraceae*_ND3007_group	Associated with disease progression	16S rRNA gene sequencing	Yes	Wu et al. (2020b)
13	Decreased expression of *Paraprevotella*, *Pseudobutyrivibrio*, and increased levels of *Collinsella stercoris*	Discriminates CKD patients from controls even in early stages	16S rRNA gene sequencing of fecal samples	Yes	Wu et al. (2020b)
14	*Ruminococcus bromii*, fecal hydroquinone, L-cystine, and 12-keto-tetrahydro-LTB4	Differentiates mild, moderate, and ESKD patients from healthy controls	Metagenomics and untargeted LC–MS/MS metabolomics of fecal and serum samples	Yes	Wang et al. (2023)
15	Vanillic acid, N-hydroxy-isoleucine, 2-aminobenzoic acid, and picolinic acid	Significantly upregulated in ESKD patients compared to CKD patients	Chemical isotope labeling liquid chromatography-mass spectrometry metabolomics of serum samples	Yes	Dahabiyeh et al. (2023)

eGFR, estimated glomerular filtration rate; IS, indoxyl sulfate; p-CS, p-cresol sulfate; ESKD, end-stage kidney disease; CKD, chronic kidney disease.

## 5 Gut microbiota and metabolites targeted interventions against CKD progression in the gut-brain-kidney axis

**Figure 3:**
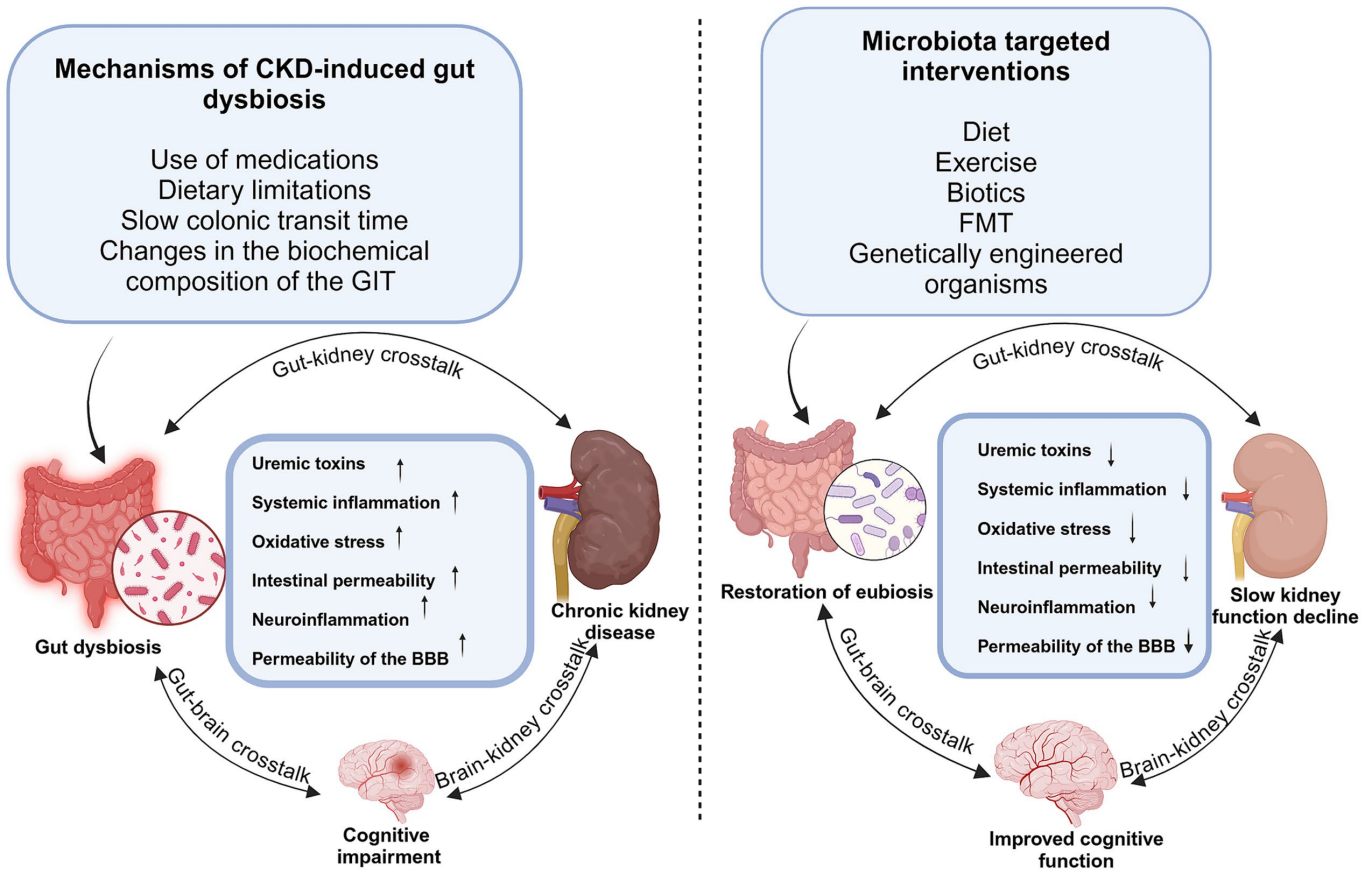
Impact of gut microbiota dysbiosis/eubiosis on the kidney and brain in CKD. Gut microbiota dysbiosis induced by medications, dietary restrictions, and slow colonic transit leads to increased production of uremic toxin precursors, such as indoles and p-cresols. Their accumulation contributes to systemic inflammation, oxidative stress, and disruption of both gut and blood–brain barriers, exacerbating kidney dysfunction and promoting neuroinflammation. Microbiota-targeted interventions such as biotics, diet, and fecal microbiota transplantation enhance SCFA production, reduce inflammation and toxin levels, restore barrier integrity, and improve renal and cognitive function in CKD.

Gut dysbiosis is implicated in the progression of a range of diseases, including cardiovascular disease, type 2 diabetes, neurodegenerative conditions, and CKD, among others, via mechanisms that are yet to be completely understood. Several pathologies that affect the brain and kidneys have been associated with dysbiosis of the gut microbiota, and compared to the general population, individuals with CKD have a higher propensity for cognitive impairment (Drew et al., 2019). This would imply that therapeutic strategies directed at the gut microbiota may prove effective for treating CKD and its related complications. Studies reveal a bidirectional cause-effect relationship between CKD and gut dysbiosis, and gut microbial metabolism is a vital source of uremic toxins. A significant influx of undigested proteins into the distal intestine as a result of impaired protein absorption encourages the overgrowth of proteolytic bacteria (Sorensen, 1965), which in turn causes an increase in protein fermentation clinically manifesting in protein-energy wasting, cardiovascular diseases, neurological syndromes, and CKD progression (Figure 3). In CKD patients, alterations in the gut microbiota and metabolite wield significant consequences as metabolites generally demonstrated to promote health, chiefly short-chain fatty acids (SCFAs), are reduced. At the same time, uremic toxins, such as indoles, ammonia, and TMAO, accumulate, thus enhancing CKD development and progression (Vaziri et al., 2016; Ramezani et al., 2016; Magliocca et al., 2022). Since the accumulation of uremic toxins in CKD patients engenders various neurological complications, treatments that target the significant reduction of these metabolites not only significantly delay CKD progression but also greatly improve cognitive function in these patients (Faucher et al., 2023). Many studies have shown that the production of uremic toxins could be reduced by selectively reducing the levels of proteolytic bacteria in the gut while simultaneously increasing saccharolytic bacteria (Ramezani et al., 2016). Many studies have embraced microbiota-targeted intervention in modulating gut microbiota and their metabolites. Many of these therapeutic strategies applied in CKD management have focused mainly on lifestyle changes (including dietary patterns and exercise), biotics intervention (probiotics, prebiotics, and synbiotics), and FMT (Figure 3).

### 5.1 Mechanisms of action

#### 5.1.1 Reduction of uremic toxins

Impaired protein absorption in CKD patients causes an increase in levels of undigested or unabsorbed proteins entering the colon, creating conditions in the gut that promote the growth of proteolytic bacterial species (Bammens et al., 2003; Evenepoel et al., 2009). Subsequently, at the distal part of the colon, tyrosine, phenylalanine, and tryptophan amino acids from endogenous, dietary, or microbial metabolism (Koppe et al., 2018) are utilized by bacteria for growth, metabolizing them to various end products, including indoles and phenols, which serve as precursors for the formation of cardio- and neuro-toxic uremic toxins (Krukowski et al., 2023). As kidney function deteriorates, these toxins accumulate in the blood, causing many deleterious effects on body tissues and organs, thereby contributing to kidney damage and related complications such as cardiovascular disease and neurological disorders (Krukowski et al., 2023; Kim et al., 2022; Vanholder et al., 2014). By improving dysbiosis, gut microbiota targeted intervention has shown promising outcomes in significantly reducing levels of these toxins in CKD patients, which, in turn, reduces the rate of occurrence of CKD-associated complications, and further studies that focus on this outcome are currently being explored.

#### 5.1.2 Enhancement of gut and blood–brain barrier integrity

Recent studies have shown that a vicious cycle of inflammation and oxidative stress is created at the renal level by the influx of uremic toxins and urea into the gastrointestinal lumen via the enterohepatic cycle, which exerts a selective pressure that encourages the excessive growth of bacterial species that produce urease, uricase, indole, and p-cresol-forming enzymes with a reduction in SCFAs-producing species (Vaziri et al., 2013; Levy et al., 2016). Bacterial urease further hydrolyzes these diffused urea to ammonia and ammonium hydroxide, thereby causing an increase in the pH of the gut lumen and altering the gut microbiota balance (Kang, 1993; Sumida et al., 2023). This imbalance of the gut microbiota and continued exposure of the colonic epithelial cells to urea is harmful to intestinal epithelial barrier function as it aids the disruption of the mucus layer and lowers the expression of tight junction proteins (Vaziri et al., 2013; Krukowski et al., 2023). Subsequent increases in the permeability of the intestinal barrier in CKD patients promote the translocation of bacterial products of intestinal origin, such as LPS, uremic toxins, and cytokines, into the systemic circulation, resulting in local and systemic inflammation, as well as oxidative stress associated with CKD (Krukowski et al., 2023; Cigarran Guldris et al., 2017; Rukavina Mikusic et al., 2020). Further accumulation of uremic toxins, which leads to uremic syndrome, can, among others, cause neurotoxicity, disruption in the BBB integrity, oxidative stress, neuroinflammation, and disturbance of brain neurotransmitter amino acids balance (Hamed, 2019).

However, SCFAs, predominantly butyrate, can enhance gut barrier integrity, thereby reducing intestinal permeability and systemic inflammation. This, by implication, protects the BBB from injury with concomitant reduction of neuroinflammation and oxidative stress. SCFAs, especially butyrate, strengthen the integrity of the gut barrier, reducing intestinal permeability and systemic inflammation. This, in turn, protects the BBB and reduces neuroinflammatory responses (Dalile et al., 2019). For instance, the probiotic *F. prausnitzii* administration to CKD mice significantly lowered the abundance of toxin-producing *Desulfovibrio* and *Collinsella*. In contrast, butyrate-producers *Faecalibacterium* and *Roseburia* were increased (Li et al., 2022). Another probiotic *Lactobacillus casei* Zhang significantly upregulated the Bacteroidetes population, a prominent butyrate producer in CKD treated mice (Zhu et al., 2021). Both probiotic bacteria significantly upregulated the expression of tight junction proteins compared to untreated mice and, thus, improved intestinal barrier function in the treated mice.

#### 5.1.3 Modulation of neuroinflammation and oxidative stress

Probiotics, prebiotics, and synbiotics modulate the production of inflammatory cytokines (e.g., IL-6, TNF-α) and oxidative stress markers, which are elevated in CKD and neurodegenerative diseases. By reducing systemic inflammation and oxidative stress, these interventions can slow the progression of kidney function decline (Chen C. et al., 2023; Liu et al., 2022).

#### 5.1.4 Production of neurotransmitters

The gut microbiota functions as an endocrine organ by producing various hormones and neurotransmitters that impact intestinal endocrine activity with the potential to regulate kidney function. Examples are acetylcholine (produced by *Lactobacillus* and *Bacillus*), gamma-aminobutyric acid (GABA) (produced by *Bifidobacterium* and *Lactobacillus*), serotonin (produced by Bifidobacterium, *Lactobacillus*, *Lactococcus*, *Enterococcus*, *Escherichia*, *Streptococcus*) catecholamine (produced by *Escherichia*, *Lactococcus*, *Bacillus*, *Lactobacillus*, and *Saccharomyces*), and tryptamine (produced by *Ruminococcus* and *Clostridium*) (Onal et al., 2019; Jazani et al., 2019; Afsar et al., 2016b). The potential of these neurotransmitters to play vital roles in the modulation of sodium homeostasis and blood pressure with impact on CKD progression has been reported (Onal et al., 2019; Fujimura et al., 1999; Skov, 2014; Skov et al., 2013; Wierema et al., 1997).

### 5.2 Lifestyle modifications in CKD management

#### 5.2.1 Dietary interventions

Limiting the intake of sodium, protein, phosphorus, and potassium is generally recommended in patients with CKD, while foods rich in fiber, vitamins, and minerals are greatly advised (Yang et al., 2018; Mafra et al., 2021). As a result, adequate care should be taken in exploring high-fiber foods, as low-potassium-containing high-fiber foods for CKD patients are the goal. By lowering phosphorus load, metabolic acidosis, and uremic toxin production, a low-protein diet (LPD) may improve CKD-associated symptoms and slow renal function deterioration (Kalantar-Zadeh et al., 2020; Kalantar-Zadeh et al., 2022). This further reduces bacterial proteolytic fermentation associated with Western diets, encouraging saccharolytic fermentation by gut bacteria and increasing SCFA production, which offers numerous health benefits to the host (Krukowski et al., 2023; Rukavina Mikusic et al., 2020). Thus, better dietary habits could positively impact CKD and its associated complications. Food consumption may impact the interlinked underlying factors associated with CKD, including inflammation, oxidative stress, mitochondrial dysfunction, and gut dysbiosis (Stenvinkel et al., 2020). Increased intake of fruits and vegetables has been linked with reduced mortality in hemodialysis patients (Saglimbene et al., 2019). This could be attributed to lower phosphorus bioavailability and lesser uremic toxins from vegetable proteins (Black et al., 2018; Joshi et al., 2021; Kandouz et al., 2016). Since all compounds present in food possess some functional attributes that may be beneficial for human health, foodome, a concept also known as food-intrinsic metabolome, has been proposed to be introduced as part of the “food as medicine” (FAM) approach for the treatment of CKD (Mafra et al., 2021).

Reduction in the rate of decrease of eGFR as well as levels of C-reactive protein, IL-6, IS, and serum cholesterol is found to be lower in CKD patients fed with a higher fiber diet, >25 g/day, compared to the group with a lower fiber intake, <25 g/day, in the 18-months follow-up period of the 157 patients that participated in the study. A high-fiber diet is also shown to be negatively correlate with the risk of cardiovascular disease in these CKD patients (Lu et al., 2017). Another meta-analysis of 16 controlled trials of low protein diet in non-dialysis-dependent CKD patients revealed that diets with restricted protein intake (<0.8 g/kg/day) were associated with higher serum bicarbonate levels, lower phosphorus levels, lower azotemia, lower rates of progression to ESKD, and a trend toward lower rates of all-cause death, as against diets with protein intake of >0.8 g/kg/day. Further findings revealed that very-low-protein diets (protein intake <0.4 g/kg/day) were associated with more remarkable preservation of kidney function and reduction in the rate of progression to ESKD. Interestingly, there were no reports of malnutrition, and the risk of protein-energy wasting and cachexia remained minimal in the patients (Rhee et al., 2018). Some researchers have also explored the combination of low-protein diets with probiotics. The clinical trial (ProLowCKD), a single-center, double-blind, placebo-controlled, randomized trial, was conducted to determine whether the association between LPD, made up of a protein intake of 0.6-g/kg of body weight or less, energy and vegetable-enriched, and sodium and phosphorus-depleted, and a mixture of the probiotics *Bifidobacterium longum* and *Lactobacillus reuteri* was effective at reducing traditional uremic, microbiota-derived, and proatherogenic toxins in 60 patients with advanced CKD that were not on dialysis. LPD significantly reduced blood urea nitrogen, total cholesterol content, and triglycerides after 2 months of intake. Further supplementation of the LPD with the selected probiotics for another 3 months in 57 selected patients countered an increase in the serum levels of IS, lipoprotein-associated phospholipase (A2 Lp-PLA2), and total cholesterol in the probiotics group compared to the placebo group. CKD patients, especially those on dialysis, are at a significantly increased risk of suffering from a stroke. Besides the traditional predisposing factors, such as the presence of diabetes, hypertension, and dyslipidemia, the accumulation of toxins in the blood of these patients is strongly associated with its occurrence. The build-up of the uremic toxin TMAO is generated from phosphatidylcholine, derived mainly from egg yolks and carnitine, mainly found in red meat (Spence, 2021). As a result, limited intake of proteins such as egg yolks and red meat, in fact, LPD in general, is proposed to improve the quality of life of CKD patients as uremic toxins are reduced and will, in effect, lower the incidence of stroke in CKD patients. A slight reduction in the levels of p-CS was also observed. Interestingly, supplementing LPD with these probiotics reduced diuretics and antihypertensive medications in the probiotics group (De Mauri et al., 2022). The meta-analysis study of Chiavaroli et al. (2015), with a total of 14 trials involving a 143 participants with CKD, revealed that dietary fiber supplementation significantly reduced the levels of serum urea and creatinine in CKD patients.

#### 5.2.2 Physical activity

CKD patients are usually known to possess poor levels of general physical activity. This results in reduced neuromuscular, cardiorespiratory, and physical functioning, ultimately leading to low quality of life and, consequently, causing an increase in morbidity and mortality during the course of the disease (Wilkinson et al., 2021a, 2021b). Many studies have documented the beneficial effects of regular exercise on both organs and the resultant whole-person aftereffects in CKD patients. Rossi et al. (2014) reported a significant improvement in physical capacity and quality of life in stages 3 to 4 CKD patients who, in addition to their usual care, received a guided renal rehabilitation exercise (twice weekly for 12 weeks). This group outperformed the group that only had the usual care treatment regimen. A study investigating a 6-month aerobic exercise intervention program in stage 4 CKD patients recorded significant improvement in health-related quality of life and kidney-related functions in the intervention group compared to the control group (Uchiyama et al., 2021). A meta-analysis including 18 randomized controlled trials with 817 patients showed that exercise training improves vascular function, thereby reducing the risk of cardiovascular events in patients with CKD (Wang H. et al., 2022). The interesting study of Xiong et al. (2022) demonstrated differences in the physical and mental health of CKD patients represented by physical component summary (PCS) and mental component summary (MCS), respectively, based on the number of their daily walking steps. Reports from the study revealed that CKD patients who walked between 7,000 and 12,000 steps daily had the highest PCS and MCS scores compared to patients who walked > 12,000 and < 7,000 daily steps. However, compared to patients with 7,000 to 12,000 daily steps count, those with >12,000 daily steps had a significantly lower MCS score, while the <7,000 daily steps patients had the lowest scores for both components. It is, therefore, possible that high health-related quality of life is associated with CKD patients with 7,000 to 12,000 daily walking steps. A recent meta-analysis that evaluated the impact of exercise on CKD patients who are not on dialysis with a focus on a variety of health indicators reported enhancements in resting heart, aerobic capacity, waist circumference, triglycerides, glycosylated hemoglobin, levels of IL-6, functional ability, and quality of life (Traise et al., 2024). The vascular system in individuals with CKD is greatly impacted by exercise training because it improves the vascular milieu by lowering oxidative stress, arterial stiffness, and systemic inflammation (Michou et al., 2024). A recent systematic review and meta-analysis revealed that exercise had a small but statistically significant impact on cognitive ability across all investigated CKD stages, with aerobic exercise being particularly beneficial (Bradshaw et al., 2024). As CKD progresses, chronic overactivation of the SNS, which raises the risk of cardiovascular disease in CKD patients, occurs. As a result, Jeong et al. (2023) also reported that aerobic exercise is very useful in improving cognitive function in CKD patients. They posited that aerobic exercise training in CKD patients might reduce SNS resting activity and vascular stiffness. Their findings revealed that aerobic exercise training provides neurovascular benefits for CKD patients as a 12-week cycling exercise showed robust efficacy in lowering the levels of resting muscle sympathetic nerve activity and aortic wave reflection determined via augmentation index, which, over time, increased in the control group.

### 5.3 Biotics intervention

The use of probiotics, prebiotics, and synbiotics in the treatment of CKD has recently gained increased awareness among renal healthcare practitioners. Some mechanisms via which biotics exert their therapeutic action include modulation of the gut microbiota by restoring balance, improving the gut-barrier integrity, and reducing inflammation and oxidative stress in CKD and neurodegenerative conditions. Some have also been efficacious in reducing the generation of uremic toxins (Hida et al., 1996), which are majorly implicated in the progression of CKD and neurodegenerative diseases.

#### 5.3.1 Probiotics

Probiotics are live microorganisms that confer health benefits on the host when administered in the right amounts (Hill et al., 2014). Using the American Gut Project database and fecal microbial data collected from a Chinese population, Li et al. showed a consistent decline in the level of the probiotic, *F. prausnitzii*, a potent butyrate producer, in CKD patients. They found that administering *F. prausnitzii* to CKD mice orally lowered renal inflammation and dysfunction and reduced the serum levels of various uremic toxins. *F. prausnitzii* considerably reduced the levels of two microbiota-derived uremic toxins p-CS, and TMAO, and one uremic toxin independent of the microbiota, guanidinosuccinic acid, in the serum of the patients. Additionally, the probiotic enhanced the gut microbial ecology and intestinal integrity. It was reported that these beneficial effects occurred through the action of *F. paustnitzii*’s metabolite, butyrate, and renal GPR (G protein-coupled receptor)-43 receptor (Li et al., 2022). Orally administering *L. casei* Zhang in CKD mice remedied gut microbiota dysbiosis caused by bilateral renal ischemia–reperfusion (I/R), mitigated kidney injury, and delayed its advancement to CKD. The action of this probiotic was attributed to its ability to increase the levels of SCFAs (butyrate and propionate) via nicotinamide metabolism in the serum and kidney, with reduced renal inflammation and decreased damage to the renal tubular epithelial cells as the resultant effect (Zhu et al., 2021). Moreover, compared to the placebo group, *L. casei* Zhang significantly reduced serum cystatin C and parathyroid hormone levels in non-dialytic CKD patients (stages 3 to 5) after 3 months of administration. The urine albumin-to-creatinine ratio was also markedly increased in the placebo group, with a mild increase in the probiotic-treated group. Subsequent follow-ups with the participants revealed that the amplitude of the increase in serum creatinine was lower, and the rate of decline of eGFR was much slower in the probiotic-treated group than in the placebo group. Thus, *L. casei* Zhang can slow down renal function decline in the tested CKD stages (Zhu et al., 2021). Although Lobun Forte and Renadyl, which are commercially available probiotics, effectively improved the quality of life in patients with stage 3–4 CKD with favorable safety profile, their modulation of uremic toxins, markers of renal function, oxidative biomarkers, and eGFR were different. While both significantly decreased the serum levels of IS, only Renadyl significantly reduced the level of p-CS, with both showing no significant effect for IAA and inflammatory markers IL-6 and TNF-α. These differences were reported for all parameters investigated (Kalidindi et al., 2024). Administration of *Bifidobacterium longum* subsp. *longum* BL21 was reported to modulate gut microbiota dysbiosis in CKD rats. This probiotic intervention enhanced the richness and diversity of key microbiota taxa, particularly *Helicobacter* and *Barnesiella*, with a profound reduction in serum uremic toxins IS, IAA, and TMAO levels. Although a significant lowering of serum p-CS and p-cresol glucuronide concentrations was not observed, the authors reported an apparent downward decline in the treated rats (Dong et al., 2024). *Lactobacillus* mix (Lm) comprising *Lactobacillus paracasei* and *Lactobacillus plantarum* strongly improved kidney function in CKD rats by reducing kidney injury and fibrotic-related proteins. A significant decrease in oxidative stress levels and inflammation was also observed. Lm reversed gut dysbiosis and restored the abundance of commensal bacteria genera (*Faecalibaculum*, *Coriobacteriaceae* UCG 002, *Lactococcus*, *Negativibacillus*, *Turicibacter*, *Ruminiclostridium* 6, *Parasutterella*, *Eubacterium xylanophilum* group, *Ruminococcaceae* UCG 010, and *Staphylococcus*) which are mainly SCFAs producers resulting in enhanced intestinal barrier integrity. Serum levels of IS and p-CS were significantly lowered, and data obtained indicated that the Lm-low dose group (10^7^ CFUs) demonstrated greater efficacy in mitigating CKD progression and restoring the gut microbiota balance as against the Lm-high dose group (10^9^ CFUs) (Huang et al., 2021). It is paramount that these probiotics’ efficacy, especially in reducing uremic toxins by modulating the gut microbiota, be further investigated in CKD patients. However, findings from probiotics supplementation in managing CKD have been inconsistent. Reduction in levels of particular uremic toxins is not always observed with different probiotics, and even one meta-analysis study reported that probiotics had no significant effect on the levels of p-CS in CKD patients (Chen et al., 2022). Properly designed experimental studies and clinical trials that address these inconsistencies are urgently needed.

#### 5.3.2 Prebiotics

Prebiotics, now known as any substrate that is selectively utilized by host microorganisms granting a health benefit (Gibson et al., 2017) have been reported to confer more consistent benefits on the host than probiotics (Mafra et al., 2021). They are known to cause a shift in bacterial metabolism from proteolytic to predominantly saccharolytic fermentation patterns in CKD patients. Administration of oligofructose-enriched inulin (p-inulin) in 15 patients with CKD in a 3-phase pilot trial revealed a marked alteration in the gut microbiota composition with an increase in abundance of *Bifidobacterium* and *Anaerostipes*. Although metabolite composition found in the stool and urine of study subjects differed significantly across study phases, an abundance of microbial metabolites derived from saccharolysis was significant (Sohn et al., 2024). Administration of resistant maltodextrin improved gut barrier integrity and remarkably increased both the relative and combined abundance of commensals (*Akkermansia*, *Bifidobacterium*, *Roseburia*, and *Lactobacillus*) in CKD rats compared to inulin and chitosan oligosaccharide (Anegkamol et al., 2023).

#### 5.3.3 Synbiotics

Synbiotics, a mixture containing live microorganisms and substrate(s) selectively utilized by host microorganisms that confer health benefits on the host (Swanson et al., 2020), have been employed to improve the diversity and richness of a dysbiotic gut, favor saccharolytic metabolism, reduce serum levels of free IS, improve intestinal permeability and alleviate constipation (Celano et al., 2023; Cosola et al., 2021). Administration of NatuREN G®, an innovative synbiotics mixture comprising *Bifidobacterium animalis*, BLC1 (10^9^ cells), *Lacticaseibacillus casei*, LC4P1 (10^9^ cells), fructooligosaccharides (2.5 g), inulin (2.5 g), quercetin (640 mg), resveratrol (230 mg), and proanthocyanidins (13 mg) to stages IIIb-IV CKD patients for 3 months reveal an increase in the ratio of Firmicutes/Bacteriodetes in the synbiotic group (Celano et al., 2023). NatuREN G^®^ also significantly reduced free serum IS in CKD patients (stage 3b to 4) after 2 months of administration (Cosola et al., 2021). Another group reported that the synbiotics supplements, which contained 100 mg Lactol probiotic comprised of *Lactobacillus* coagulants and Fructo-oligosaccharides (FOS) administered to hemodialysis patients in a randomized, double-blind, placebo-controlled trial significantly reduced total iron binding capacity in the synbiotic group. Hence, iron-deficiency-induced anemia in these patients improved (Kooshki et al., 2023). Significant reductions in serum levels of total IS, p-CS, IL-6, and malondialdehyde (as markers of inflammation and oxidative stress, respectively) were observed in 30 patients with ESKD undergoing hemodialysis treatment after an 8-week administration of synbiotics containing 2 × 10^11^ (CFU)/g of *Lactobacillus acidophilus* La-14 and FOS (Kuskunov et al., 2023). The synbiotic comprising two pills, each containing 4 × 10^9^ CFU of *Lactobacillus acidophilus* CBT LA1, 4 × 10^9^ of *Lactobacillus casei* CBT LC5, and 8 × 10^9^ of *Bifidobacterium lactis* CBT BL3 together with 1.6 g of inulin was administered to non-dialyzed CKD patients in a 12-week randomized, double-blind, placebo-controlled study. The synbiotic intervention was found to significantly modify the gut microbiota, increase the relative abundance of *Lactobacillus*, *Bifidobacteria*, and *Subdoligranulum* genera, substantially lower the serum level of IS, improve eGFR, and reduce the level of high-sensitivity C-reactive protein in the treated group. Besides increased flatulence in 2 patients from the intervention group, no other safety concerns were reported (Mitrović et al., 2023). Lydia et al. (2022) observed that although a two-month synbiotic intervention consisting of 5×10^9^ CFU *Lactobacillus acidophilus* and *Bifidobacterium longum* and 60 mg of FOS in a double-blinded randomized controlled clinical trial involving hemodialysis patients with gastrointestinal complaints had a substantial effect in improving constipation and general quality of life in the synbiotic group, it did not reduce the levels of IS in their serum compared to the placebo group.

While numerous synbiotic formulations have demonstrated potential in modulating gut microbiota and reducing uremic toxin levels in patients with CKD, their translation into routine clinical practice remains limited. Among these, Renadyl^®^, developed by Kibow Biotech, stands out as one of the few commercially available synbiotic products that has undergone clinical evaluation in both CKD and ESKD populations (Ranganathan et al., 2010; Natarajan et al., 2014; Pechenyak, 2013). This formulation comprises three probiotic strains, *Streptococcus thermophilus* (KB19), *Lactobacillus acidophilus* (KB27), and *Bifidobacterium longum* (KB31) each delivered at a concentration of 45 billion colony-forming units (CFU), together with the prebiotic fibers xylo-oligosaccharide and inulin. Renadyl^®^ is designed to act through the gut-kidney axis, where the probiotic strains metabolize uremic toxins in the colon, thereby facilitating their diffusion from the bloodstream and subsequent fecal elimination.

Clinical studies have indicated that Renadyl^®^ is safe and may reduce serum levels of select uremic toxins, with standardized dosing protocols ranging from 90 to 270 billion CFUs per day (Pechenyak, 2013). Although marketed as a dietary supplement rather than a pharmaceutical, it is available over the counter and is generally well tolerated by patients. Nonetheless, the absence of approval from the U. S. Food and Drug Administration (FDA) as a therapeutic agent, alongside issues of cost, accessibility, and long-term adherence, may limit its broader clinical integration. Ongoing research is warranted to establish its long-term efficacy, cost-effectiveness, and potential inclusion in standard CKD management protocols.

#### 5.3.4 Observations from meta-analysis studies of biotics intervention

A meta-analysis study of dialysis patients by Chen C. et al. (2023), which included 18 randomized controlled trials, revealed that supplementation of probiotics, prebiotics, and synbiotics in CKD patients could significantly lower C-reactive protein, IL-6, and IS and improved levels of high-density lipoprotein cholesterol in comparison to the control group, but had no substantial effect on p-CS, low-density lipoprotein, cholesterol, TNF-α, triglyceride, albumin, hemoglobin, total cholesterol, phosphorus, calcium, or uric acid in the serum of the patients (Chen C. et al., 2023). Another meta-analysis study of 12 randomized controlled trials involving non-dialysis patients revealed that biotic supplements did not significantly reduce the serum creatinine levels compared to the placebo group (Liu F. et al., 2023). A meta-analysis that included 23 studies with 842 participants of randomized controlled trials that evaluated any biotic intervention in stage 3 to 4 CKD and also ESKD patients revealed that the biotics improved total antioxidative capacity and malondialdehyde and reduced the levels of IL-6. Some uremic toxins, such as p-CS and IS, were also lowered by the supplements. Prebiotics alone reduced levels of blood urea nitrogen and serum creatinine. However, significantly positive effects of the biotics intervention on other markers of renal function, such as eGFR, serum albumin, C-reactive protein, IAA levels, and lipid metabolites, were not observed (Liu et al., 2022). A meta-analysis study of CKD patients (both dialytic and non-dialytic) involving 14 randomized controlled trials showed that compared to the placebo group, biotics supplementation had no statistically significant effect on the levels of circulating IS, but a substantial decrease was associated with circulating p-CS concentration. Findings from the subgroup analysis also revealed that although prebiotics and synbiotics significantly reduced the concentration of circulating p-CS, probiotics did not (Chen et al., 2022).

### 5.4 Genetically engineered bacteria

Genetically engineering bacteria for enhanced abilities has increased the therapeutic options available for any microbiota-targeted intervention strategy. In their pioneering work, Prakash and Chang (1996) introduced a novel strategy by encapsulating genetically engineered *Escherichia coli* DH5 cells within semipermeable polymeric microcapsules. This design allowed the controlled release of metabolites, such as urea, while preventing the release of genetically modified cells into host tissues, thus mitigating safety concerns. Their study, conducted in a rat model of uremia, demonstrated that the encapsulated bacteria effectively reduced elevated plasma urea levels without systemic exposure to the engineered cells, presenting a promising strategy for urea reduction in CKD. Building on this, Cai et al. explored the therapeutic potential of uricase-expressing genetically engineered *E. coli* for hyperuricemia, a condition often linked to CKD. By secreting active uricase, these engineered bacteria successfully lowered serum uric acid (SUA) levels in rats, offering a novel solution to a gap in current treatment strategies, which primarily focus on reducing uric acid production rather than enhancing its excretion. This microbial strategy holds significant potential for managing both hyperuricemia and its related complications, such as gout and CKD, by improving the excretion of uric acid, which is crucial in preventing urate crystal deposition and subsequent renal inflammation (Cai et al., 2020). Additionally the team of Lubkowicz et al. (2024) in their recent study, genetically engineered *E. coli* Nissle 1917 (*Ec*N) to efficiently convert L-tyrosine, the substrate from which p-cresol, the precursor for the uremic toxin p-CS is derived from to p-coumaric acid which is a non-toxic compound via tyrosine ammonia lyase. By initiating the breakdown of L-tyrosine in the proximal region of the small intestine with consequent reduction of the quantity reaching the colon and by competing with the native gut microbiota in the colon implicated in p-cresol generation from L-tyrosine, the therapeutic strain exerts its activity in CKD patients. These studies show that genetically engineered microorganisms can offer precise and low-risk treatments for complex metabolic diseases, opening up new possibilities in microbiology-based therapies.

### 5.5 Fecal microbiota transplantation

FMT, a procedure involving the transfer of stools from a healthy donor for the purpose of modulating the gut microbiota to a more balanced state, was first applied and validated in the treatment of *Clostridium difficile* infection. The relationship between gut dysbiosis and CKD progression has been investigated using FMT from animal models of CKD, CKD patients, and healthy donors. For instance, administration of FMT from ESKD patients to renal injured germ-free mice or antibiotic-treated rats resulted in an increased production of serum uraemic toxins, exacerbated renal fibrosis and oxidative stress, while control models with FMTs from healthy donors did not show these effects (Wang et al., 2020). The application of FMT from healthy mice to adenine-induced CKD mice improved gastrointestinal symptoms and significantly reduced p-CS in CKD mice. Furthermore, there was substantial restoration of the richness of the gut microbiota in these mice, as demonstrated by the increased alpha diversity observed (Barba et al., 2020). Although there was no change in kidney function in this study, it further validates the possibility of FMT use in managing CKD. A recent clinical trial study that evaluated alterations in CKD patients with diabetes and/or hypertension presenting with stages 2, 3, and 4 of the disease treated with either FMT or placebo capsules for 6 months showed that the patients had similar responses to FMT, regardless of their CKD stage. Relative to the placebo group, FMT profoundly modulated the gut microbiota of the patients involved with a reduction in the abundance of Firmicutes and Actinobacteria and an increase in *Bacteroidetes*, *Proteobacteria,* and *Roseburia* spp. The authors also observed the maintenance of serum creatinine and urea nitrogen (markers of stable renal function) and reduced disease progression in the FMT group (Arteaga-Muller et al., 2024). Further studies of FMT application in CKD management, especially in restoring gut dysbiosis, reducing uremic toxins, inflammation, oxidative stress, and other markers of renal function, are needed. It is also essential that at all times, safety measures regarding FMT use are ensured to prevent the transfer of harmful pathogens to recipients being evaluated.

### 5.6 Personalized medicine and holistic approaches

The increasing knowledge of the gut-brain-kidney axis offers the potential for personalized treatments, especially in CKD patients. Probiotics can promote health, but their effects differ in individuals, particularly those with altered gut ecology arising from specific diseases such as CKD. There is also the likelihood that a probiotic bacteria’s action may depend on the individual’s CKD stage (Tian et al., 2022). Treatments tailored toward the needs of individual patients based on their CKD stage and microbiome profiles via the application of different -omics approaches such as microbiome analysis, metabolomics, metagenomics, metaproteomics, nutrigenomics, and others enable the application of suitable dietary requirements, physical activity, and/or biotic supplementation that will be most beneficial to them via modulation of the microbiota, reduction of uremic toxins and inflammation, and slowing CKD progression ultimately leading to an improvement in their cognitive health and overall quality of life.

## 6 Challenges and future directions

Clinical trials that employ the lifestyle changes and microbiota-based approaches discussed above are usually carried out in small cohorts. Larger sample sizes of CKD patients across the different stages of CKD are needed to make more robust evidence-based research conclusions. Longer follow-up times primarily focused on pertinent clinical outcomes, including survival, are also vital, and research with treatment application is strongly advocated. Studies continue to demonstrate wide-range benefits of exercise across CKD and cognitive function; however, the implementation of exercise still needs to be improved (Michou et al., 2024; Bradshaw et al., 2024; Jeong et al., 2023). It is hoped that with increasing global evidence, high-quality clinical studies, and sustained clinician and patient engagement, exercise programs will become better prioritized in the nephrology field. Again, certain probiotics may be very effective *in vitro* but may not show the same *in vivo*efficacy *in vivo*, limiting their use in translational studies. This, may in part, be improved by genetically engineering the probiotics involved. Genetic engineering of probiotic bacteria for enhanced efficiency in significantly lowering uremic toxins is still in its infancy. Therefore, rational and properly designed experiments that utilize this technology in CKD management are vital. This also requires further testing of the genetically engineered EcN (Traise et al., 2024) to determine its efficacy *in vivo*.

At present, there are no treatments established to prevent cognitive decline in CKD patients, and the limited range of medications available for cognitive impairment only have moderate activity (Yiannopoulou et al., 2019). Moreover, most microbiota-based intervention strategies do not have standard doses and duration of administration in CKD patients (Tian et al., 2022). However, therapeutic approaches that are multi-faceted, adequately combining conventional CKD medications, oral sorbent AST 120 (Evenepoel et al., 2009; Liu et al., 2018), with lifestyle and microbiota-based treatments specially tailored toward the specific needs of the patients could be more efficacious in the management of CKD and related complications. It is also worth mentioning that many CKD studies that apply microbiota intervention report that patients show very minimal to no side effects to their use. This indicates a considerable safety profile associated with them and furthers why their use in CKD management is strongly advocated.

## 7 Conclusion

The influence of the gut microbiota and metabolites in the gut-brain-kidney axis, especially in relation to CKD, is a novel research area currently gaining increasing interest among researchers of renal health and the gut microbiome. Extensive progress in -omics technologies has unveiled the impact of various lifestyles and gut-microbiota/metabolites modulation in CKD patients compared to healthy controls and patients not exposed to these therapeutic measures. Moreover, the bidirectional communication pathways between the gut-kidney, brain-kidney, and gut-brain suggest that any regulation of one pathway will inevitably influence the others. Physical activity, dietary changes, and other microbiota-targeted intervention strategies discussed in this review are extremely beneficial in CKD management as they offer a non-invasive approach to the reduction of uremic toxins, enhancement of the intestinal barrier integrity and that of the BBB, lowering inflammation and oxidative stress, SCFAs generation, modulation of neurotransmitter levels, and immune responses that ultimately slow kidney function decline, decreasing the occurrence of associated complications, and greatly improving the quality of lives of affected individuals. Studies on gut microbiota-targeted approaches have also revealed biomarkers that can be utilized for both early diagnostic and prognostic purposes in cases of CKD. However, further studies, that focus on optimizing specific strains to determine appropriate doses and best integrative personalized formulations for individual patients, and also long-term clinical trials that aim to assess and validate the efficacy of these interventions in diverse populations are needed. Nevertheless, increasing evidence in this field emphasizes the importance of the gut microbiota in the maintenance of kidney and brain health, implying that further targeting of the gut microbiota via various means holds potential for the improvement of such chronic disease conditions as CKD and neurodegenerative disorders.

## References

[ref1] Adesso S. Magnus T. Cuzzocrea S. Campolo M. Rissiek B. Paciello O.. (2017). Indoxyl sulfate affects glial function increasing oxidative stress and Neuroinflammation in chronic kidney disease: interaction between astrocytes and microglia. Front. Pharmacol. 8:370. doi: 10.3389/fphar.2017.00370, PMID: 28659803 PMC5466960

[ref2] Afsar B. Sag A. A. Yalcin C. E. Kaya E. Siriopol D. Goldsmith D.. (2016a). Brain-kidney cross-talk: definition and emerging evidence. Eur. J. Intern. Med. 36, 7–12. doi: 10.1016/j.ejim.2016.07.032, PMID: 27531628

[ref3] Afsar B. Vaziri N. D. Aslan G. Tarim K. Kanbay M. (2016b). Gut hormones and gut microbiota: implications for kidney function and hypertension. J. Am. Soc. Hypertens. 10, 954–961. doi: 10.1016/j.jash.2016.10.007, PMID: 27865823

[ref4] Ahmed H. Leyrolle Q. Koistinen V. Kärkkäinen O. Layé S. Delzenne N.. (2022). Microbiota-derived metabolites as drivers of gut–brain communication. Gut Microbes 14:2102878. doi: 10.1080/19490976.2022.2102878, PMID: 35903003 PMC9341364

[ref5] Albillos A. de Gottardi A. Rescigno M. (2020). The gut-liver axis in liver disease: pathophysiological basis for therapy. J. Hepatol. 72, 558–577. doi: 10.1016/j.jhep.2019.10.003, PMID: 31622696

[ref6] Amini Khiabani S. Asgharzadeh M. Samadi Kafil H. (2023). Chronic kidney disease and gut microbiota. Heliyon 9:e18991. doi: 10.1016/j.heliyon.2023.e18991, PMID: 37609403 PMC10440536

[ref7] Anders H. J. Andersen K. Stecher B. (2013). The intestinal microbiota, a leaky gut, and abnormal immunity in kidney disease. Kidney Int. 83, 1010–1016. doi: 10.1038/ki.2012.440, PMID: 23325079

[ref8] Andrade-Oliveira V. Amano M. T. Correa-Costa M. Castoldi A. Felizardo R. J. F. de Almeida D. C.. (2015). Gut Bacteria products prevent AKI induced by ischemia-reperfusion. J. Am. Soc. Nephrol. 26, 1877–1888. doi: 10.1681/ASN.2014030288, PMID: 25589612 PMC4520159

[ref9] Anegkamol W. Kamkang P. Hunthai S. Kaewwongse M. Taweevisit M. (2023). The usefulness of resistant maltodextrin and chitosan oligosaccharide in management of gut leakage and microbiota in chronic kidney disease. Nutrients 15:3363. doi: 10.3390/nu15153363, PMID: 37571302 PMC10420640

[ref10] Arteaga-Muller G. Y. Flores-Treviño S. Bocanegra-Ibarias P. Robles-Espino D. Garza-González E. Fabela-Valdez G. C.. (2024). Changes in the progression of chronic kidney disease in patients undergoing fecal microbiota transplantation. Nutrients 16:1109. doi: 10.3390/nu16081109, PMID: 38674803 PMC11055146

[ref11] Balint L. Socaciu C. Socaciu A. I. Vlad A. Gadalean F. Bob F.. (2023a). Metabolite profiling of the gut–renal–cerebral Axis reveals a particular pattern in early diabetic kidney disease in T2DM patients. Int. J. Mol. Sci. 24:6212. doi: 10.3390/ijms24076212, PMID: 37047187 PMC10094272

[ref12] Balint L. Socaciu C. Socaciu A. I. Vlad A. Gadalean F. Bob F.. (2023c). Quantitative, targeted analysis of gut microbiota derived metabolites provides novel biomarkers of early diabetic kidney disease in type 2 diabetes mellitus patients. Biomol. Ther. 13:1086. doi: 10.3390/biom13071086, PMID: 37509122 PMC10377254

[ref13] Balint L. Socaciu C. Socaciu A. I. Vlad A. Gadalean F. Bob F.. (2023b). Metabolites potentially derived from gut microbiota associated with podocyte, proximal tubule, and renal and cerebrovascular endothelial damage in early diabetic kidney disease in T2DM patients. Meta 13:893. doi: 10.3390/metabo13080893, PMID: 37623837 PMC10456401

[ref14] Bammens B. Verbeke K. Vanrenterghem Y. Evenepoel P. (2003). Evidence for impaired assimilation of protein in chronic renal failure. Kidney Int. 64, 2196–2203. doi: 10.1046/j.1523-1755.2003.00314.x, PMID: 14633143

[ref15] Barba C. Soulage C. O. Caggiano G. Glorieux G. Fouque D. Koppe L. (2020). Effects of fecal microbiota transplantation on composition in mice with CKD. Toxins 12:741. doi: 10.3390/toxins12120741, PMID: 33255454 PMC7761367

[ref16] Bartochowski P. Gayrard N. Bornes S. Druart C. Argilés A. Cordaillat-Simmons M.. (2022). Gut–kidney Axis investigations in animal models of chronic kidney disease. Toxins 14:626. doi: 10.3390/toxins14090626, PMID: 36136564 PMC9502418

[ref17] Bercik P. Park A. J. Sinclair D. Khoshdel A. Lu J. Huang X.. (2011). The anxiolytic effect of *Bifidobacterium longum* NCC3001 involves vagal pathways for gut-brain communication. Neurogastroenterol. Motil. 23, 1132–1139. doi: 10.1111/j.1365-2982.2011.01796.x, PMID: 21988661 PMC3413724

[ref18] Black A. P. Anjos J. S. Cardozo L. Carmo F. L. Dolenga C. J. Nakao L. S.. (2018). Does low-protein diet influence the uremic toxin serum levels from the gut microbiota in nondialysis chronic kidney disease patients? J. Ren. Nutr. 28, 208–214. doi: 10.1053/j.jrn.2017.11.007, PMID: 29439931

[ref19] Bobot M. Thomas L. Moyon A. Fernandez S. McKay N. Balasse L.. (2020). Uremic toxic blood-brain barrier disruption mediated by AhR activation leads to cognitive impairment during experimental renal dysfunction. J. Am. Soc. Nephrol. 31, 1509–1521. doi: 10.1681/ASN.2019070728, PMID: 32527975 PMC7350986

[ref20] Bolyen E. Rideout J. R. Dillon M. R. Bokulich N. A. Abnet C. C. Al-Ghalith G. A.. (2019). Reproducible, interactive, scalable and extensible microbiome data science using QIIME 2. Nat. Biotechnol. 37, 852–857. doi: 10.1038/s41587-019-0209-9, PMID: 31341288 PMC7015180

[ref21] Bradshaw E. Alejmi A. Rossetti G. D’Avossa G. Macdonald J. H. (2024). Exercise and cognitive function chronic kidney disease: a systematic review and meta-analysis of efficacy and harms. Clin. J. Am. Soc. Nephrol. 19, 1461–1472. doi: 10.2215/CJN.0000000000000533 39083357 PMC11556908

[ref22] Bugnicourt J. M. Godefroy O. Chillon J. M. Choukroun G. Massy Z. A. (2013). Cognitive disorders and dementia in CKD. J. Am. Soc. Nephrol. 24, 353–363. doi: 10.1681/asn.2012050536, PMID: 23291474

[ref23] Buoli M. Dozio E. Caldiroli L. Armelloni S. Vianello E. Corsi Romanelli M.. (2024). Clinical factors and biomarkers associated with depressive disorders in older patients affected by chronic kidney disease (CKD): does the advanced glycation end products (AGEs)/RAGE (receptor for AGEs) system play any role? Geriatrics 9:99. doi: 10.3390/geriatrics9040099, PMID: 39195129 PMC11353441

[ref24] Cai L. Li Q. Deng Y. Liu X. Du W. Jiang X. (2020). Construction and expression of recombinant uricase-expressing genetically engineered bacteria and its application in rat model of hyperuricemia. Int. J. Mol. Med. 45, 1488–1500. doi: 10.3892/ijmm.2020.4512 32323736 PMC7138262

[ref25] Cao W. Yang Z. Liu X. Ren S. Su H. Yang B.. (2023). A kidney-brain neural circuit drives progressive kidney damage and heart failure. Signal Transduct. Target. Ther. 8:184. doi: 10.1038/s41392-023-01402-x, PMID: 37169751 PMC10175540

[ref26] Castro P. Azevedo E. Rocha I. Sorond F. Serrador J. M. (2018). Chronic kidney disease and poor outcomes in ischemic stroke: is impaired cerebral autoregulation the missing link? BMC Neurol. 18:21. doi: 10.1186/s12883-018-1025-4, PMID: 29499637 PMC5834853

[ref27] Cedillo-Flores R. Cuevas-Budhart M. A. Cavero-Redondo I. Kappes M. Ávila-Díaz M. Paniagua R. (2025). Impact of gut microbiome modulation on uremic toxin reduction in chronic kidney disease: A systematic review and network Meta-analysis. Nutrients 17:1247. doi: 10.3390/nu17071247, PMID: 40219004 PMC11990722

[ref28] Celano G. Calabrese F. M. Rocchetti M. T. Iacobellis I. Serale N. Calasso M.. (2023). *In vivo* evaluation of an innovative synbiotics on stage IIIb-IV chronic kidney disease patients. Front. Nutr. 10:1215836. doi: 10.3389/fnut.2023.1215836 37396126 PMC10311028

[ref29] Chelluboina B. Vemuganti R. (2019). Chronic kidney disease in the pathogenesis of acute ischemic stroke. J. Cereb. Blood Flow Metab. 39, 1893–1905. doi: 10.1177/0271678X19866733, PMID: 31366298 PMC6775591

[ref30] Chen Y. Y. Chen D. Q. Chen L. Liu J. R. Vaziri N. D. Guo Y.. (2019). Microbiome–metabolome reveals the contribution of gut–kidney axis on kidney disease. J. Transl. Med. 17:5. doi: 10.1186/s12967-018-1756-4, PMID: 30602367 PMC6317198

[ref31] Chen T. H. Cheng C. Y. Huang C. K. Ho Y. H. Lin J. C. (2023). Exploring the relevance between gut microbiota-metabolites pro fi le and chronic kidney disease with distinct pathogenic. Microbiol. Spectr. 11, 1–15. doi: 10.1128/spectrum.02805-22 PMC9927243 36475922

[ref32] Chen L. Shi J. Ma X. Shi D. Qu H. (2022). Effects of microbiota-driven therapy on circulating Indoxyl sulfate and P-Cresyl sulfate in patients with chronic kidney disease: A systematic review and Meta-analysis of randomized controlled trials. Adv. Nutr. 13, 1267–1278. doi: 10.1093/advances/nmab149, PMID: 34905018 PMC9340978

[ref33] Chen C. Wang J. Li J. Zhang W. Ou S. (2023). Probiotics, prebiotics, and Synbiotics for patients on Dialysis: A systematic review and Meta-analysis of randomized controlled trials. J. Ren. Nutr. 33, 126–139. doi: 10.1053/j.jrn.2022.04.001, PMID: 35452837

[ref34] Chiavaroli L. Mirrahimi A. Sievenpiper J. L. Jenkins D. J. A. Darling P. B. (2015). Dietary fiber effects in chronic kidney disease: a systematic review and meta-analysis of controlled feeding trials. Eur. J. Clin. Nutr. 69, 761–768. doi: 10.1038/ejcn.2014.237, PMID: 25387901

[ref35] Cigarran Guldris S. González Parra E. Cases Amenós A. (2017). Gut microbiota in chronic kidney disease. Nefrologia 37, 9–19. doi: 10.1016/j.nefro.2016.05.008, PMID: 27553986

[ref36] Connell E. Le Gall G. Pontifex M. G. Sami S. Cryan J. F. Clarke G.. (2022). Microbial-derived metabolites as a risk factor of age-related cognitive decline and dementia. Mol. Neurodegener. 17:43. doi: 10.1186/s13024-022-00548-6, PMID: 35715821 PMC9204954

[ref37] Cosola C. Rocchetti M. T. di Bari I. Acquaviva P. M. Maranzano V. Corciulo S.. (2021). An innovative Synbiotic formulation decreases free serum Indoxyl sulfate, small intestine permeability and ameliorates gastrointestinal symptoms in a randomized pilot trial in stage IIIb-IV CKD patients. Toxins 13:334. doi: 10.3390/toxins13050334, PMID: 34063068 PMC8147955

[ref38] Dahabiyeh L. A. Nimer R. M. Sumaily K. M. Alabdaljabar M. S. Jacob M. Sabi E. M.. (2023). Metabolomics profiling distinctively identified end-stage renal disease patients from chronic kidney disease patients. Sci. Rep. 13:6161. doi: 10.1038/s41598-023-33377-8, PMID: 37061630 PMC10105740

[ref39] Dalal R. Bruss Z. S. Sehdev J. S. (2024). Physiology, renal blood flow and filtration. Treasure Island, Florida, USA: StatPearls. 29489242

[ref40] Dalile B. Van Oudenhove L. Vervliet B. Verbeke K. (2019). The role of short-chain fatty acids in microbiota–gut–brain communication. Nat. Rev. Gastroenterol. Hepatol. 16, 461–478. doi: 10.1038/s41575-019-0157-3, PMID: 31123355

[ref41] De Mauri A. Carrera D. Bagnati M. Rolla R. Vidali M. Chiarinotti D.. (2022). Probiotics-supplemented low-protein diet for microbiota modulation in patients with advanced chronic kidney disease (ProLowCKD): results from a placebo-controlled randomized trial. Nutrients 14:1637. doi: 10.3390/nu14081637, PMID: 35458199 PMC9025298

[ref42] Dicks L. M. T. (2022). Gut bacteria and neurotransmitters. Microorganisms 10:1838. doi: 10.3390/microorganisms10091838 36144440 PMC9504309

[ref43] Diep T. N. Liu H. Yan L. J. (2025). Beneficial effects of butyrate on kidney disease. Nutrients 17:772. doi: 10.3390/nu17050772, PMID: 40077642 PMC11901450

[ref44] Dinan T. G. Cryan J. F. (2017). Brain–gut–microbiota axis — mood, metabolism and behaviour. Nat. Rev. Gastroenterol. Hepatol. 14, 69–70. doi: 10.1038/nrgastro.2016.200, PMID: 28053341

[ref45] Dong Y. Gai Z. Han M. Xu J. Zou K. (2024). Reduction in serum concentrations of uremic toxins driven by *Bifidobacterium longum* subsp. *longum* BL21 is associated with gut microbiota changes in a rat model of chronic kidney disease. Probiotics Antimicrob. Proteins. doi: 10.1007/s12602-024-10293-5 PMC12405026 38829564

[ref46] Dowling L. R. Strazzari M. R. Keely S. Kaiko G. E. (2022). Enteric nervous system and intestinal epithelial regulation of the gut-brain axis. J. Allergy Clin. Immunol. 150, 513–522. doi: 10.1016/j.jaci.2022.07.015, PMID: 36075637

[ref47] Drew D. A. Weiner D. E. Sarnak M. J. (2019). Cognitive impairment in CKD: pathophysiology, management, and prevention. Am. J. Kidney Dis. 74, 782–790. doi: 10.1053/j.ajkd.2019.05.017, PMID: 31378643 PMC7038648

[ref48] Dulam V. Katta S. Nakka V. P. (2024). Stroke and distal organ damage: exploring brain-kidney crosstalk. Neurochem. Res. 49, 1617–1627. doi: 10.1007/s11064-024-04126-8, PMID: 38376748

[ref49] Evenepoel P. Meijers B. K. I. Bammens B. R. M. Verbeke K. (2009). Uremic toxins originating from colonic microbial metabolism. Kidney Int. 76, S12–S19. doi: 10.1038/ki.2009.402, PMID: 19946322

[ref50] Evenepoel P. Poesen R. Meijers B. (2017). The gut–kidney axis. Pediatr. Nephrol. 32, 2005–2014. doi: 10.1007/s00467-016-3527-x, PMID: 27848096

[ref51] Evenepoel P. Stenvinkel P. Shanahan C. Pacifici R. (2023). Inflammation and gut dysbiosis as drivers of CKD–MBD. Nat. Rev. Nephrol. 19, 646–657. doi: 10.1038/s41581-023-00736-7, PMID: 37488276

[ref52] Faucher Q. van der Made T. K. De Lange E. Masereeuw R. (2023). Blood-brain barrier perturbations by uremic toxins: key contributors in chronic kidney disease-induced neurological disorders? Eur. J. Pharm. Sci. Off. J. Eur. Fed. Pharm. Sci. 187:106462. doi: 10.1016/j.ejps.2023.106462, PMID: 37169097

[ref53] Felizardo R. J. F. Watanabe I. K. M. Dardi P. Rossoni L. V. Câmara N. O. S. (2019). The interplay among gut microbiota, hypertension and kidney diseases: the role of short-chain fatty acids. Pharmacol. Res. 141, 366–377. doi: 10.1016/j.phrs.2019.01.019, PMID: 30639376

[ref54] Freeman W. D. Wadei H. M. (2015). A brain–kidney connection: the delicate interplay of brain and kidney physiology. Neurocrit. Care. 22, 173–175. doi: 10.1007/s12028-015-0119-8, PMID: 25672972

[ref55] Fujimura S. Shimakage H. Tanioka H. Yoshida M. Suzuki-Kusaba M. Hisa H.. (1999). Effects of GABA on noradrenaline release and vasoconstriction induced by renal nerve stimulation in isolated perfused rat kidney. Br. J. Pharmacol. 127, 109–114. doi: 10.1038/sj.bjp.0702524, PMID: 10369462 PMC1565999

[ref56] Gela Y. Y. Tesfaye W. Melese M. Getnet M. Ambelu A. Eshetu H. B.. (2024). Common mental disorders and associated factors among adult chronic kidney disease patients attending referral hospitals in Amhara regional state. Sci. Rep. 14:6812. doi: 10.1038/s41598-024-57512-1, PMID: 38514836 PMC10957902

[ref57] Gibson G. R. Hutkins R. W. Prescott S. L. (2017). The international scientific association for probiotics and prebiotics (ISAPP) consensus statement on the definition and scope of prebiotics. Nat. Rev. Gastroenterol. Hepatol. 14, 491–502. doi: 10.1038/nrgastro.2017.75 28611480

[ref58] Hamed S. A. (2019). Neurologic conditions and disorders of uremic syndrome of chronic kidney disease: presentations, causes, and treatment strategies. Expert. Rev. Clin. Pharmacol. 12, 61–90. doi: 10.1080/17512433.2019.1555468, PMID: 30501441

[ref59] Han S. K. Kim D. H. (2019). Lactobacillus mucosae and *Bifidobacterium longum* synergistically alleviate immobilization stress-induced anxiety/depression in mice by suppressing gut Dysbiosis. J. Microbiol. Biotechnol. 29, 1369–1374. doi: 10.4014/jmb.1907.07044, PMID: 31564078

[ref60] Hanscom M. Loane D. J. Shea-Donohue T. (2021). Brain-gut axis dysfunction in the pathogenesis of traumatic brain injury. J. Clin. Invest. 131:e143777. doi: 10.1172/JCI143777, PMID: 34128471 PMC8203445

[ref61] He M. Wei W. Zhang Y. Xiang Z. Peng D. Kasimumali A.. (2024). Gut microbial metabolites SCFAs and chronic kidney disease. J. Transl. Med. 22:172. doi: 10.1186/s12967-024-04974-6, PMID: 38369469 PMC10874542

[ref62] Hida M. Aiba Y. Sawamura S. Suzuki N. Satoh T. Koga Y. (1996). Inhibition of the accumulation of uremic toxins in the blood and their precursors in the feces after Oral Administration of Lebenin&reg;, a lactic acid Bacteria preparation, to uremic patients undergoing hemodialysis. Nephron 74, 349–355. doi: 10.1159/000189334, PMID: 8893154

[ref63] Hill C. Guarner F. Reid G. Gibson G. R. Merenstein D. J. Pot B.. (2014). The international scientific Association for Probiotics and Prebiotics consensus statement on the scope and appropriate use of the term probiotic. Nat. Rev. Gastroenterol. Hepatol. 11, 506–514. doi: 10.1038/nrgastro.2014.66, PMID: 24912386

[ref64] Huang H. Li K. Lee Y. Chen M. (2021). Preventive effects of *Lactobacillus* mixture against chronic kidney disease progression through enhancement of beneficial bacteria and downregulation of gut-derived uremic toxins. J. Agric. Food Chem. 69, 7353–7366. doi: 10.1021/acs.jafc.1c01547 34170659

[ref65] Hui Y. Zhao J. Yu Z. Wang Y. Qin Y. Zhang Y.. (2023). The role of tryptophan metabolism in the occurrence and progression of acute and chronic kidney diseases. Mol. Nutr. Food Res. 67:e2300218. doi: 10.1002/mnfr.202300218, PMID: 37691068

[ref66] Jadoul M. Aoun M. Masimango Imani M. (2024). The major global burden of chronic kidney disease. Lancet Glob. Health 12, e342–e343. doi: 10.1016/S2214-109X(24)00050-0 38365398

[ref67] Jang H. R. Gandolfo M. T. Ko G. J. Satpute S. Racusen L. Rabb H. (2009). Early exposure to germs modifies kidney damage and inflammation after experimental ischemia-reperfusion injury. Am. J. Physiol. Renal. Physiol. 297, F1457–F1465. doi: 10.1152/ajprenal.90769.2008 19675178 PMC2781336

[ref68] Jazani N. H. Savoj J. Lustgarten M. Lau W. L. Vaziri N. D. (2019). Impact of gut Dysbiosis on Neurohormonal pathways in chronic kidney disease. Diseases 7:21. doi: 10.3390/diseases7010021, PMID: 30781823 PMC6473882

[ref69] Jeong J. Sprick J. D. DaCosta D. R. Mammino K. Nocera J. R. Park J. (2023). Exercise modulates sympathetic and vascular function in chronic kidney disease. JCI Insight 8:e164221. doi: 10.1172/jci.insight.164221, PMID: 36810250 PMC9977504

[ref70] Jiang S. Xie S. Lv D. Zhang Y. Deng J. Zeng L.. (2016). A reduction in the butyrate producing species Roseburia spp. and *Faecalibacterium prausnitzii* is associated with chronic kidney disease progression. Antonie Van Leeuwenhoek 109, 1389–1396. doi: 10.1007/s10482-016-0737-y, PMID: 27431681

[ref71] Joshi S. McMacken M. Kalantar-Zadeh K. (2021). Plant-based diets for kidney disease: a guide for clinicians. Am. J. Kidney Dis. 77, 287–296. doi: 10.1053/j.ajkd.2020.10.003, PMID: 33075387

[ref72] Kalantar-Zadeh K. Joshi S. Schlueter R. Cooke J. Brown-Tortorici A. Donnelly M.. (2020). Plant-dominant low-protein diet for conservative Management of Chronic Kidney Disease. Nutrients 12:1931. doi: 10.3390/nu12071931, PMID: 32610641 PMC7400005

[ref73] Kalantar-Zadeh K. Lockwood M. B. Rhee C. M. Tantisattamo E. Andreoli S. Balducci A.. (2022). Patient-centred approaches for the management of unpleasant symptoms in kidney disease. Nat. Rev. Nephrol. 18, 185–198. doi: 10.1038/s41581-021-00518-z, PMID: 34980890

[ref74] Kalidindi R. K. Reddy C. P. K P. Kompella P. (2024). The efficacy and safety of probiotic combinations Lobun forte® versus Renadyl® in patients with chronic kidney disease: a comparative, phase IV, randomized, open-label, active-controlled, parallel study. Cureus [Internet] 16:e67987. doi: 10.7759/cureus.67987 39347194 PMC11427930

[ref75] Kandouz S. Mohamed A. S. Zheng Y. Sandeman S. Davenport A. (2016). Reduced protein bound uraemic toxins in vegetarian kidney failure patients treated by haemodiafiltration. Hemodial. Int. 20, 610–617. doi: 10.1111/hdi.12414, PMID: 27044443

[ref76] Kandpal M. Indari O. Baral B. Jakhmola S. Tiwari D. Bhandari V.. (2022). Dysbiosis of gut microbiota from the perspective of the gut–brain Axis: role in the provocation of neurological disorders. Meta 12:1064. doi: 10.3390/metabo12111064, PMID: 36355147 PMC9692419

[ref77] Kang J. Y. (1993). The gastrointestinal tract in uremia. Dig. Dis. Sci. 38, 257–268. doi: 10.1007/BF01307542, PMID: 8425438

[ref78] Kearns R. (2024). The kynurenine pathway in gut permeability and inflammation. Inflammation, 1–15. doi: 10.1007/s10753-024-02135-x, PMID: 39256304 PMC12234587

[ref79] Keefer L. (2018). Behavioural medicine and gastrointestinal disorders: the promise of positive psychology. Nat. Rev. Gastroenterol. Hepatol. 15, 378–386. doi: 10.1038/s41575-018-0001-1, PMID: 29651112

[ref80] Kim D. S. Kim S. W. Gil H. W. (2022). Emotional and cognitive changes in chronic kidney disease. Korean J. Intern. Med. 37, 489–501. doi: 10.3904/kjim.2021.492, PMID: 35249316 PMC9082446

[ref81] Kooshki A. Akbarzadeh R. Amin B. Tofighiyan T. (2023). Synbiotic supplement for treatment of iron deficiency anaemia in haemodialysis patients: a randomized controlled trial. Nephrology 28, 234–239. doi: 10.1111/nep.14149, PMID: 36745046

[ref82] Koppe L. Fouque D. Soulage C. O. (2018). The role of gut microbiota and diet on uremic retention solutes production in the context of chronic kidney disease. Toxins 10:155. doi: 10.3390/toxins10040155, PMID: 29652797 PMC5923321

[ref83] Krukowski H. Valkenburg S. Madella A. M. Garssen J. Van Bergenhenegouwen J. Overbeek S. A.. (2023). Gut microbiome studies in CKD: opportunities, pitfalls and therapeutic potential. Nat. Rev. Nephrol. 19, 87–101. doi: 10.1038/s41581-022-00647-z, PMID: 36357577

[ref84] Kuskunov T. Tilkiyan E. Doykov D. Boyanov K. Bivolarska A. Hristov B. (2023). The effect of synbiotic supplementation on uremic toxins, oxidative stress, and inflammation in hemodialysis patients — results of an uncontrolled prospective single-arm study. Medicina (Kaunas) 59:1383. doi: 10.3390/medicina59081383 37629672 PMC10456308

[ref85] Lau W. L. Kalantar-Zadeh K. Vaziri N. D. (2015). The gut as a source of inflammation in chronic kidney disease. Nephron 130, 92–98. doi: 10.1159/000381990, PMID: 25967288 PMC4485546

[ref86] Levy M. Thaiss C. A. Elinav E. (2016). Metabolites: messengers between the microbiota and the immune system. Genes Dev. 30, 1589–1597. doi: 10.1101/gad.284091.116, PMID: 27474437 PMC4973288

[ref87] Li L. Ma L. Fu P. (2017). Gut microbiota&ndash;derived short-chain fatty acids and kidney diseases. Drug Des. Devel. Ther. 11, 3531–3542. doi: 10.2147/DDDT.S150825, PMID: 29270002 PMC5729884

[ref88] Li H. B. Xu M. L. Xu X. D. Tang Y. Y. Jiang H. L. Li L.. (2022). *Faecalibacterium prausnitzii* Attenuates CKD via Butyrate-Renal GPR43 Axis. Circ. Res. 131, e120–e134. doi: 10.1161/CIRCRESAHA.122.320184, PMID: 36164984 PMC9588706

[ref89] Lin X. Yu Z. Liu Y. Li C. Hu H. Hu J.. (2025). Gut–X axis. iMeta 4:e270. doi: 10.1002/imt2.270 40027477 PMC11865426

[ref90] Liu W. Huang J. Liu T. Hu Y. Shi K. Zhou Y.. (2023). Changes in gut microbial community upon chronic kidney disease. PLoS One 18:e0283389. doi: 10.1371/journal.pone.0283389 36952529 PMC10035866

[ref91] Liu F. Liu Y. Lv X. Lun H. (2023). Effects of prebiotics, probiotics and synbiotics on serum creatinine in non-dialysis patients: a meta-analysis of randomized controlled trials. Ren. Fail. 45:2152693. doi: 10.1080/0886022X.2022.2152693, PMID: 36636981 PMC9848283

[ref92] Liu W. C. Tomino Y. Lu K. C. (2018). Impacts of Indoxyl sulfate and p-cresol sulfate on chronic kidney disease and mitigating effects of AST-120. Toxins 10:367. doi: 10.3390/toxins10090367, PMID: 30208594 PMC6162782

[ref93] Liu J. Zhong J. Yang H. Wang D. Zhang Y. Yang Y.. (2022). Biotic supplements in patients with chronic kidney disease: meta-analysis of randomized controlled trials. J. Ren. Nutr. 32, 10–21. doi: 10.1053/j.jrn.2021.08.005, PMID: 34666930 PMC9793596

[ref94] Liyanage T. Toyama T. Hockham C. Ninomiya T. Perkovic V. Woodward M.. (2022). Prevalence of chronic kidney disease in Asia: a systematic review and analysis. BMJ Glob. Health 7:e007525. doi: 10.1136/bmjgh-2021-007525, PMID: 35078812 PMC8796212

[ref95] Lu L. Huang Y. F. Wang M. Q. Chen D. X. Wan H. Wei L. B.. (2017). Dietary fiber intake is associated with chronic kidney disease (CKD) progression and cardiovascular risk, but not protein nutritional status, in adults with CKD. Asia Pac. J. Clin. Nutr. 26, 598–605. doi: 10.6133/apjcn.072016.08, PMID: 28582807

[ref96] Lu R. Kiernan M. C. Murray A. Rosner M. H. Ronco C. (2015). Kidney–brain crosstalk in the acute and chronic setting. Nat. Rev. Nephrol. 11, 707–719. doi: 10.1038/nrneph.2015.131, PMID: 26281892

[ref97] Lubkowicz D. Hava D. L. Lewis K. Isabella V. M. (2024). Rational engineering of *Escherichia coli* Nissle 1917 as live biotherapeutic to degrade uremic toxin precursors. ACS Synth. Biol. 13, 1077–1084. doi: 10.1021/acssynbio.3c00686, PMID: 38588591

[ref98] Lv J. C. Zhang L. X. (2019). Prevalence and disease burden of chronic kidney disease. Adv. Exp. Med. Biol. 1165, 3–15. doi: 10.1007/978-981-13-8871-2_1 31399958

[ref99] Lydia A. Indra T. A. Rizka A. Abdullah M. (2022). The effects of synbiotics on indoxyl sulphate level, constipation, and quality of life associated with constipation in chronic haemodialysis patients: a randomized controlled trial. BMC Nephrol. 23:259. doi: 10.1186/s12882-022-02890-9 35869437 PMC9308250

[ref100] Mafra D. Borges N. A. Lindholm B. Shiels P. G. Evenepoel P. Stenvinkel P. (2021). Food as medicine: targeting the uraemic phenotype in chronic kidney disease. Nat. Rev. Nephrol. 17, 153–171. doi: 10.1038/s41581-020-00345-8, PMID: 32963366

[ref101] Magliocca G. Mone P. Di Iorio B. R. Heidland A. Marzocco S. (2022). Short-chain fatty acids in chronic kidney disease: focus on inflammation and oxidative stress regulation. Int. J. Mol. Sci. 23:5354. doi: 10.3390/ijms23105354, PMID: 35628164 PMC9140893

[ref102] Mao Z. H. Gao Z. X. Liu D. W. Liu Z. S. Wu P. (2023). Gut microbiota and its metabolites - molecular mechanisms and management strategies in diabetic kidney disease. Front. Immunol. 14:1124704. doi: 10.3389/fimmu.2023.1124704, PMID: 36742307 PMC9896007

[ref103] Marano G. Mazza M. Lisci F. M. Ciliberto M. Traversi G. Kotzalidis G. D.. (2023). The microbiota–gut–brain Axis: Psychoneuroimmunological insights. Nutrients 15:1496. doi: 10.3390/nu15061496, PMID: 36986226 PMC10059722

[ref104] Margolis K. G. Cryan J. F. Mayer E. A. (2021). The microbiota-gut-brain Axis: from motility to mood. Gastroenterology 160, 1486–1501. doi: 10.1053/j.gastro.2020.10.066, PMID: 33493503 PMC8634751

[ref105] Mayer E. A. Nance K. Chen S. (2022). The gut–brain Axis. Annu. Rev. Med. 73, 439–453. doi: 10.1146/annurev-med-042320-014032, PMID: 34669431

[ref106] Mazumder M. K. Paul R. Bhattacharya P. Borah A. (2019). Neurological sequel of chronic kidney disease: from diminished acetylcholinesterase activity to mitochondrial dysfunctions, oxidative stress and inflammation in mice brain. Sci. Rep. 9:3097. doi: 10.1038/s41598-018-37935-3, PMID: 30816118 PMC6395638

[ref107] Meijers B. K. I. Evenepoel P. (2011). The gut-kidney axis: indoxyl sulfate, p-cresyl sulfate and CKD progression. Nephrol. Dial. Transplant. 26, 759–761. doi: 10.1093/ndt/gfq818, PMID: 21343587

[ref108] Michou V. Tsamos G. Vasdeki D. Deligiannis A. Kouidi E. (2024). Unraveling of molecular mechanisms of cognitive frailty in chronic kidney disease: how exercise makes a difference. J. Clin. Med. 13:5698. doi: 10.3390/jcm13195698, PMID: 39407758 PMC11476541

[ref109] Mitrović M. Stanković-Popović V. Tolinački M. Golić N. Bajić S. S. Veljović K.. (2023). The impact of synbiotic treatment on the levels of gut-derived uremic toxins, inflammation, and gut microbiome of chronic kidney disease patients-a randomized trial. J. Ren. Nutr. 33, 278–288. doi: 10.1053/j.jrn.2022.07.008, PMID: 35995418

[ref110] Natarajan R. Pechenyak B. Vyas U. Ranganathan P. Weinberg A. Liang P.. (2014). Randomized controlled trial of strain-specific probiotic formulation (Renadyl) in Dialysis patients. Biomed. Res. Int. 2014, 1–9. doi: 10.1155/2014/568571, PMID: 25147806 PMC4132402

[ref111] Needham B. D. Funabashi M. Adame M. D. Wang Z. Boktor J. C. Haney J.. (2022). A gut-derived metabolite alters brain activity and anxiety behaviour in mice. Nature 602, 647–653. doi: 10.1038/s41586-022-04396-8, PMID: 35165440 PMC9170029

[ref112] Noel S. Mohammad F. White J. Lee K. Gharaie S. Rabb H. (2021). Gut microbiota-immune system interactions during acute kidney injury. Kidney360 2, 528–531. doi: 10.34067/KID.0006792020, PMID: 35369013 PMC8785987

[ref113] Onal E. M. Afsar B. Covic A. Vaziri N. D. Kanbay M. (2019). Gut microbiota and inflammation in chronic kidney disease and their roles in the development of cardiovascular disease. Hypertens. Res. 42, 123–140. doi: 10.1038/s41440-018-0144-z, PMID: 30504819

[ref114] Pechenyak B. (2013). Dose escalation, safety and impact of a strain-specific probiotic (Renadyl™) on stages III and IV chronic kidney disease patients. J. Nephrol. Ther. 3:141. doi: 10.4172/2161-0959.1000141

[ref115] Pires L. González-Paramás A. M. Heleno S. A. Calhelha R. C. (2024). The role of gut microbiota in the Etiopathogenesis of multiple chronic diseases. Antibiotics 13:392. doi: 10.3390/antibiotics13050392, PMID: 38786121 PMC11117238

[ref116] Pluznick J. L. (2016). Gut microbiota in renal physiology: focus on short-chain fatty acids and their receptors. Kidney Int. 90, 1191–1198. doi: 10.1016/j.kint.2016.06.033, PMID: 27575555 PMC5123942

[ref117] Prakash S. Chang T. M. S. (1996). Microencapsulated genetically engineered live *E. coli* DH5 cells administered orally to maintain normal plasma urea level in uremic rats. Nat. Med. 2, 883–887. doi: 10.1038/nm0896-883, PMID: 8705857

[ref118] Ramezani A. Massy Z. A. Vanholder R. Raj D. S. (2016). Role of the gut microbiome in uremia: a potential therapeutic target. Am. J. Kidney Dis. 67:483. doi: 10.1053/j.ajkd.2015.09.027, PMID: 26590448 PMC5408507

[ref119] Ranganathan N. Friedman E. A. Tam P. Rao V. Ranganathan P. Dheer R. (2009). Probiotic dietary supplementation in patients with stage 3 and 4 chronic kidney disease: a 6-month pilot scale trial in Canada. Curr. Med. Res. Opin. 25, 1919–1930. doi: 10.1185/03007990903069249, PMID: 19558344

[ref120] Ranganathan N. Ranganathan P. Friedman E. A. Joseph A. Delano B. Goldfarb D. S.. (2010). Pilot study of probiotic dietary supplementation for promoting healthy kidney function in patients with chronic kidney disease. Adv. Ther. 27, 634–647. doi: 10.1007/s12325-010-0059-9, PMID: 20721651

[ref121] Rhee C. M. Ahmadi S. f. Kovesdy C. P. Kalantar-zadeh K. (2018). Low-protein diet for conservative management of chronic kidney disease: a systematic review and meta-analysis of controlled trials. J. Cachexia. Sarcopenia Muscle 9, 235–245. doi: 10.1002/jcsm.12264, PMID: 29094800 PMC5879959

[ref122] Romaní-Pérez M. Bullich-Vilarrubias C. López-Almela I. Liébana-García R. Olivares M. Sanz Y. (2021). The microbiota and the gut–brain Axis in controlling food intake and energy homeostasis. Int. J. Mol. Sci. 22:5830. doi: 10.3390/ijms22115830, PMID: 34072450 PMC8198395

[ref123] Rooks M. G. Garrett W. S. (2016). Gut microbiota, metabolites and host immunity. Nat. Rev. Immunol. 16, 341–352. doi: 10.1038/nri.2016.42, PMID: 27231050 PMC5541232

[ref124] Rossi A. P. Burris D. D. Lucas F. L. Crocker G. A. Wasserman J. C. (2014). Effects of a renal rehabilitation exercise program in patients with CKD: a randomized, controlled trial. Clin. J. Am. Soc. Nephrol. 9, 2052–2058. doi: 10.2215/CJN.11791113, PMID: 25414318 PMC4255415

[ref125] Rossing P. Caramori M. L. Chan J. C. N. Heerspink H. J. L. Hurst C. Khunti K.. (2022). KDIGO 2022 clinical practice guideline for diabetes management in chronic kidney disease. Kidney Int. 102, S1–S127. doi: 10.1016/j.kint.2022.06.008, PMID: 36272764

[ref126] Rovin B. H. Ayoub I. M. Chan T. M. Liu Z. H. Mejía-Vilet J. M. Floege J. (2024). Kdigo 2024 clinical practice guideline for the management of lupus nephritis. Kidney Int. 105, S1–S69. doi: 10.1016/j.kint.2023.09.002 38182286

[ref127] Rukavina Mikusic N. L. Kouyoumdzian N. M. Choi M. R. (2020). Gut microbiota and chronic kidney disease: evidences and mechanisms that mediate a new communication in the gastrointestinal-renal axis. Pflügers Arch. 472, 303–320. doi: 10.1007/s00424-020-02352-x, PMID: 32064574

[ref128] Rysz J. Franczyk B. Ławiński J. Olszewski R. Ciałkowska-Rysz A. Gluba-Brzózka A. (2021). The impact of CKD on uremic toxins and gut microbiota. Toxins 13:252. doi: 10.3390/toxins13040252, PMID: 33807343 PMC8067083

[ref129] Saglimbene V. M. Wong G. Ruospo M. Palmer S. C. Garcia-Larsen V. Natale P.. (2019). Fruit and vegetable intake and mortality in adults undergoing maintenance hemodialysis. Clin. J. Am. Soc. Nephrol. 14, 250–260. doi: 10.2215/CJN.08580718, PMID: 31738182 PMC6390927

[ref130] Saliba A. Debnath S. Tamayo I. Tumova J. Maddox M. Singh P.. (2024). Quinolinic acid links kidney injury to brain toxicity. bioRxiv. doi: 10.1101/2024.05.07.592801 PMC11949017 39946208

[ref131] Salminen A. (2023). Activation of aryl hydrocarbon receptor (AhR) in Alzheimer’s disease: role of tryptophan metabolites generated by gut host-microbiota. J. Mol. Med. 101, 201–222. doi: 10.1007/s00109-023-02289-5, PMID: 36757399 PMC10036442

[ref132] Shiao C. C. Wu P. C. Huang T. M. Lai T. S. Yang W. S. Wu C. H.. (2015). Long-term remote organ consequences following acute kidney injury. Crit. Care 19:438. doi: 10.1186/s13054-015-1149-5, PMID: 26707802 PMC4699348

[ref133] Sinha S. Lin G. Ferenczi K. (2021). The skin microbiome and the gut-skin axis. Clin. Dermatol. 39, 829–839. doi: 10.1016/j.clindermatol.2021.08.021, PMID: 34785010

[ref134] Skov J. (2014). Effects of GLP-1 in the kidney. Rev. Endocr. Metab. Disord. 15, 197–207. doi: 10.1007/s11154-014-9287-7, PMID: 24791975

[ref135] Skov J. Dejgaard A. Frøkiær J. Holst J. J. Jonassen T. Rittig S.. (2013). Glucagon-like peptide-1 (GLP-1): effect on kidney hemodynamics and renin-angiotensin-aldosterone system in healthy men. J. Clin. Endocrinol. Metab. 98, E664–E671. doi: 10.1210/jc.2012-3855, PMID: 23463656

[ref136] Sohn M. B. Gao B. Kendrick C. Srivastava A. Isakova T. Gassman J. J.. (2024). Targeting gut microbiome with prebiotic in patients with CKD: the TarGut-CKD study. Kidney Int Rep. 9, 671–685. doi: 10.1016/j.ekir.2023.12.017, PMID: 38481512 PMC10927482

[ref137] Sokol H. Pigneur B. Watterlot L. Lakhdari O. Bermúdez-Humarán L. G. Gratadoux J. J.. (2008). *Faecalibacterium prausnitzii* is an anti-inflammatory commensal bacterium identified by gut microbiota analysis of Crohn disease patients. Proc. Natl. Acad. Sci. 105, 16731–16736. doi: 10.1073/pnas.0804812105, PMID: 18936492 PMC2575488

[ref138] Sorensen L. B. (1965). Role of the intestinal tract in the elimination of uric acid. Arthritis Rheum. 8, 694–706. doi: 10.1002/art.1780080429, PMID: 5859543

[ref139] Spence J. D. (2021). Reducing the risk of stroke in patients with impaired renal function: nutritional issues. J. Stroke Cerebrovasc. Dis. 30:105376. doi: 10.1016/j.jstrokecerebrovasdis.2020.105376, PMID: 33214054

[ref140] Stenvinkel P. Meyer C. J. Block G. A. Chertow G. M. Shiels P. G. (2020). Understanding the role of the cytoprotective transcription factor nuclear factor erythroid 2-related factor 2—lessons from evolution, the animal kingdom and rare progeroid syndromes. Nephrol. Dial. Transplant. 35, 2036–2045. doi: 10.1093/ndt/gfz120, PMID: 31302696 PMC7716811

[ref141] Stevens P. E. Ahmed S. B. Carrero J. J. Foster B. Francis A. Hall R. K.. (2024). KDIGO 2024 clinical practice guideline for the evaluation and management of chronic kidney disease. Kidney Int. 105, S117–S314. doi: 10.1016/j.kint.2023.10.018, PMID: 38490803

[ref142] Stier C. T. Rocha R. Chander P. N. (2005). Effect of aldosterone and MR blockade on the brain and the kidney. Heart Fail. Rev. 10, 53–62. doi: 10.1007/s10741-005-2349-x, PMID: 15947892

[ref143] Strid H. Simrén M. Stotzer P. O. Ringström G. Abrahamsson H. Björnsson E. S. (2003). Patients with chronic renal failure have abnormal small intestinal motility and a high prevalence of small intestinal bacterial overgrowth. Digestion 67, 129–137. doi: 10.1159/000071292, PMID: 12853724

[ref144] Su X. Gao Y. Yang R. (2022). Gut microbiota-derived tryptophan metabolites maintain gut and systemic homeostasis. Cells 11:2296. doi: 10.3390/cells11152296, PMID: 35892593 PMC9330295

[ref145] Sumida K. Pierre J. F. Yuzefpolskaya M. Colombo P. C. Demmer R. T. Kovesdy C. P. (2023). Gut microbiota-targeted interventions in the Management of Chronic Kidney Disease. Semin. Nephrol. 43:151408. doi: 10.1016/j.semnephrol.2023.151408, PMID: 37619529 PMC10783887

[ref146] Sun C. Y. Li J. R. Wang Y. Y. Lin S. Y. Ou Y. C. Lin C. J.. (2021). Indoxyl sulfate caused behavioral abnormality and neurodegeneration in mice with unilateral nephrectomy. Aging 13, 6681–6701. doi: 10.18632/aging.202523, PMID: 33621199 PMC7993681

[ref147] Swanson K. S. Gibson G. R. Hutkins R. Reimer R. A. Reid G. Verbeke K.. (2020). The international scientific Association for Probiotics and Prebiotics (ISAPP) consensus statement on the definition and scope of synbiotics. Nat. Rev. Gastroenterol. Hepatol. 17, 687–701. doi: 10.1038/s41575-020-0344-2, PMID: 32826966 PMC7581511

[ref148] Teng Y. Mu J. Xu F. Zhang X. Sriwastva M. K. Liu Q. M.. (2022). Gut bacterial isoamylamine promotes age-related cognitive dysfunction by promoting microglial cell death. Cell Host Microbe 30, 944–960.e8. doi: 10.1016/j.chom.2022.05.005, PMID: 35654045 PMC9283381

[ref149] Teubner W. Meinl W. Florian S. Kretzschmar M. Glatt H. (2007). Identification and localization of soluble sulfotransferases in the human gastrointestinal tract. Biochem. J. 404, 207–215. doi: 10.1042/BJ20061431, PMID: 17335415 PMC1868804

[ref150] Tian N. Li L. Ng J. K. C. Li P. K. T. (2022). The potential benefits and controversies of probiotics use in patients at different stages of chronic kidney disease. Nutrients 14:4044. doi: 10.3390/nu14194044, PMID: 36235699 PMC9571670

[ref151] Tidgren B. Hjemdahl P. (1989). Renal responses to mental stress and epinephrine in humans. Am. J. Physiol. Ren. Physiol. 257, F682–F689. 10.1152/ajprenal.1989.257.4.F682 2679146

[ref152] Traise A. Dieberg G. Pearson M. J. Smart N. A. (2024). The effect of exercise training in people with pre-dialysis chronic kidney disease: a systematic review with meta-analysis. J. Nephrol. 37, 2063–2098. doi: 10.1007/s40620-024-02081-9, PMID: 39417982 PMC11649798

[ref153] Turin T. C. James M. Ravani P. Tonelli M. Manns B. J. Quinn R.. (2013). Proteinuria and rate of change in kidney function in a community-based population. J. Am. Soc. Nephrol. 24, 1661–1667. doi: 10.1681/ASN.2012111118, PMID: 23833255 PMC3785273

[ref154] Uchiyama K. Adachi K. Muraoka K. Nakayama T. Oshida T. Yasuda M. (2021). Home-based aerobic exercise and resistance training for severe chronic kidney disease: a randomized controlled trial. J. Cachexia. Sarcopenia Muscle 12, 1789–1802. doi: 10.1002/jcsm.12775, PMID: 34554649 PMC8718025

[ref155] Vallianou N. G. Kounatidis D. Panagopoulos F. Evangelopoulos A. Stamatopoulos V. Papagiorgos A.. (2023). Gut microbiota and its role in the brain-gut-kidney Axis in hypertension. Curr. Hypertens. Rep. 25, 367–376. doi: 10.1007/s11906-023-01263-3, PMID: 37632662

[ref156] Vanholder R. Schepers E. Pletinck A. Nagler E. V. Glorieux G. (2014). The uremic toxicity of Indoxyl sulfate and p-Cresyl sulfate: A systematic review. J. Am. Soc. Nephrol. 25, 1897–1907. doi: 10.1681/ASN.2013101062, PMID: 24812165 PMC4147984

[ref157] Vaziri N. D. Wong J. Pahl M. Piceno Y. M. Yuan J. DeSantis T. Z.. (2013). Chronic kidney disease alters intestinal microbial flora. Kidney Int. 83, 308–315. doi: 10.1038/ki.2012.345, PMID: 22992469

[ref158] Vaziri N. D. Zhao Y. y. Pahl M. V. (2016). Altered intestinal microbial flora and impaired epithelial barrier structure and function in CKD: the nature, mechanisms, consequences and potential treatment. Nephrol. Dial. Transplant. 31, 737–746. doi: 10.1093/ndt/gfv095 25883197

[ref159] Voroneanu L. Burlacu A. Brinza C. Covic A. Balan G. G. Nistor I.. (2023). Gut microbiota in chronic kidney disease: from composition to modulation towards better outcomes—A systematic review. J. Clin. Med. 12:1948. doi: 10.3390/jcm12051948, PMID: 36902734 PMC10003930

[ref160] Wang H. Ainiwaer A. Song Y. Qin L. Peng A. Bao H.. (2023). Perturbed gut microbiome and fecal and serum metabolomes are associated with chronic kidney disease severity. Microbiome 11:3. doi: 10.1186/s40168-022-01443-4, PMID: 36624472 PMC9827681

[ref161] Wang S. Dong Z. (2016). Environmental hit on a genetic basis in polycystic kidney disease. Am. J. Physiol. Ren. Physiol. 311, F1358–F1359. doi: 10.1152/ajprenal.00452.2016 PMC5210190 27558561

[ref162] Wang W. Jiang S. Xu C. Tang L. Liang Y. Zhao Y.. (2022). Interactions between gut microbiota and Parkinson’s disease: the role of microbiota-derived amino acid metabolism. Front. Aging Neurosci. 14:14. doi: 10.3389/fnagi.2022.976316, PMID: 36408101 PMC9667037

[ref163] Wang S. Lv D. Jiang S. Jiang J. Liang M. Hou F.. (2019). Quantitative reduction in short-chain fatty acids, especially butyrate, contributes to the progression of chronic kidney disease. Clin. Sci. 133, 1857–1870. doi: 10.1042/CS20190171, PMID: 31467135

[ref164] Wang M. Pan W. Xu Y. Zhang J. Wan J. Jiang H. (2022). Microglia-mediated Neuroinflammation: A potential target for the treatment of cardiovascular diseases. J. Inflamm. Res. 15, 3083–3094. doi: 10.2147/JIR.S350109, PMID: 35642214 PMC9148574

[ref165] Wang H. Xie D. Wu L. Zhao L. (2022). Association of exercise with vascular function in patients with CKD: a meta-analysis of randomized controlled trials. Front Med (Lausanne) 9:904299. doi: 10.3389/fmed.2022.904299 35872793 PMC9299368

[ref166] Wang X. Yang S. Li S. Zhao L. Hao Y. Qin J.. (2020). Aberrant gut microbiota alters host metabolome and impacts renal failure in humans and rodents. Gut 69, 2131–2142. doi: 10.1136/gutjnl-2019-319766, PMID: 32241904 PMC7677483

[ref167] Wierema T. K. Houben A. J. de Leeuw P. W. (1997). Acetylcholine-induced vasodilatation in the human hypertensive kidney: inhibition by muscarinic receptor antagonism. J. Hypertens. 15, 1649–1651. doi: 10.1097/00004872-199715120-00067, PMID: 9488217

[ref168] Wilkinson T. J. Clarke A. L. Nixon D. G. D. Hull K. L. Song Y. Burton J. O.. (2021a). Prevalence and correlates of physical activity across kidney disease stages: an observational multicentre study. Nephrol. Dial. Transplant. 36, 641–649. doi: 10.1093/ndt/gfz235 31725147

[ref169] Wilkinson T. J. Mcadams-demarco M. Bennett P. N. Wilund K. Team K. L. Affairs M. C.. (2021b). Advances in exercise therapy in predialysis chronic kidney disease, hemodialysis, peritoneal dialysis, and kidney transplantation. Curr. Opin. Nephrol. Hypertens. 29, 471–479. doi: 10.1097/MNH.0000000000000627 PMC7526394 32701595

[ref170] Wiredu Ocansey D. K. Hang S. Yuan X. Qian H. Zhou M. Valerie Olovo C.. (2023). The diagnostic and prognostic potential of gut bacteria in inflammatory bowel disease. Gut Microbes 15:2176118. doi: 10.1080/19490976.2023.2176118, PMID: 36794838 PMC9980661

[ref171] Wong J. Piceno Y. M. DeSantis T. Z. Pahl M. Andersen G. L. Vaziri N. D. (2014). Expansion of urease- and uricase-containing, indole- and p-cresol-forming and contraction of short-chain fatty acid-producing intestinal microbiota in ESRD. Am. J. Nephrol. 39, 230–237. doi: 10.1159/000360010, PMID: 24643131 PMC4049264

[ref172] Wu I. W. Gao S. S. Chou H. C. Yang H. Y. Chang L. C. Kuo Y. L.. (2020a). Integrative metagenomic and metabolomic analyses reveal severity-specific signatures of gut microbiota in chronic kidney disease. Theranostics 10, 5398–5411. doi: 10.7150/thno.41725, PMID: 32373220 PMC7196299

[ref173] Wu I. W. Lin C. Y. Chang L. C. Lee C. C. Chiu C. Y. Hsu H. J.. (2020b). Gut microbiota as diagnostic tools for mirroring disease progression and circulating Nephrotoxin levels in chronic kidney disease: discovery and validation study. Int. J. Biol. Sci. 16, 420–434. doi: 10.7150/ijbs.37421, PMID: 32015679 PMC6990903

[ref174] Xie Z. Tong S. Chu X. Feng T. Geng M. (2022). Chronic kidney disease and cognitive impairment: the kidney-brain Axis. Kidney Dis. 8, 275–285. doi: 10.1159/000524475, PMID: 36157262 PMC9386403

[ref175] Xiong J. Peng H. Yu Z. Chen Y. Pu S. Li Y.. (2022). Daily walking dose and health-related quality of life in patients with chronic kidney disease. J. Ren. Nutr. 32, 710–717. doi: 10.1053/j.jrn.2022.01.015, PMID: 35134535

[ref176] Yan Q. Liu M. Xie Y. Lin Y. Fu P. Pu Y.. (2024). Kidney-brain axis in the pathogenesis of cognitive impairment. Neurobiol. Dis. 200:106626. doi: 10.1016/j.nbd.2024.106626, PMID: 39122123

[ref177] Yang T. Richards E. M. Pepine C. J. Raizada M. K. (2018). The gut microbiota and the brain–gut–kidney axis in hypertension and chronic kidney disease. Nat. Rev. Nephrol. 14, 442–456. doi: 10.1038/s41581-018-0018-2, PMID: 29760448 PMC6385605

[ref178] Yiannopoulou K. G. Anastasiou A. I. Kyrozis A. Anastasiou I. P. (2019). Donepezil treatment for Alzheimer’s disease in chronic dialysis patients. Case Rep. Nephrol. Dial. 9, 126–136. doi: 10.1159/000502682, PMID: 31616673 PMC6787415

[ref179] Zhang Y. W. Song P. R. Wang S. C. Liu H. Shi Z. M. Su J. C. (2024). Diets intervene osteoporosis via gut-bone axis. Gut Microbes 16:2295432. doi: 10.1080/19490976.2023.2295432, PMID: 38174650 PMC10773645

[ref180] Zhao Z. Ning J. Bao X. qi Shang M. Ma J.. (2021). Fecal microbiota transplantation protects rotenone-induced Parkinson’s disease mice via suppressing inflammation mediated by the lipopolysaccharide-TLR4 signaling pathway through the microbiota-gut-brain axis. Microbiome 9:226. doi: 10.1186/s40168-021-01107-9, PMID: 34784980 PMC8597301

[ref181] Zhao J. Zhang Q. Cheng W. Dai Q. Wei Z. Guo M.. (2023). Heart–gut microbiota communication determines the severity of cardiac injury after myocardial ischaemia/reperfusion. Cardiovasc. Res. 119, 1390–1402. doi: 10.1093/cvr/cvad023, PMID: 36715640 PMC10262181

[ref182] Zhou X. Ji S. Chen L. Liu X. Deng Y. You Y.. (2024). Gut microbiota dysbiosis in hyperuricaemia promotes renal injury through the activation of NLRP3 inflammasome. Microbiome 12:109. doi: 10.1186/s40168-024-01826-9, PMID: 38907332 PMC11191305

[ref183] Zhou T. Zhao J. Ma Y. He L. Ren Z. Yang K.. (2024). Association of cognitive impairment with the interaction between chronic kidney disease and depression: findings from NHANES 2011–2014. BMC Psychiatry 24:312. doi: 10.1186/s12888-024-05769-1, PMID: 38658863 PMC11044494

[ref184] Zhu H. Cao C. Wu Z. Zhang H. Sun Z. Wang M.. (2021). The probiotic *L. casei* Zhang slows the progression of acute and chronic kidney disease. Cell Metab. 33, 1926–1942e8. doi: 10.1016/j.cmet.2021.06.014 34270930

